# Purkinje Cell-Specific Knockout of Tyrosine Hydroxylase Impairs Cognitive Behaviors

**DOI:** 10.3389/fncel.2020.00228

**Published:** 2020-07-29

**Authors:** Timothy M. Locke, Hirofumi Fujita, Avery Hunker, Shelby S. Johanson, Martin Darvas, Sascha du Lac, Larry S. Zweifel, Erik S. Carlson

**Affiliations:** ^1^Department of Psychiatry and Behavioral Sciences, University of Washington, Seattle, WA, United States; ^2^Department of Pharmacology, University of Washington, Seattle, WA, United States; ^3^Department of Otolaryngology—Head and Neck Surgery, Johns Hopkins University, Baltimore, MD, United States; ^4^Geriatric Research, Education and Clinical Center, Veteran’s Affairs Medical Center, Puget Sound, Seattle, WA, United States; ^5^Department of Pathology, University of Washington, Seattle, WA, United States; ^6^Department of Neurology, Johns Hopkins University, Baltimore, MD, United States; ^7^Department of Neuroscience, Johns Hopkins University, Baltimore, MD, United States

**Keywords:** catecholamine, cerebellum, cognition, purkinje cell, dopamine

## Abstract

Tyrosine hydroxylase (Th) expression has previously been reported in Purkinje cells (PCs) of rodents and humans, but its role in the regulation of behavior is not understood. Catecholamines are well known for facilitating cognitive behaviors and are expressed in many regions of the brain. Here, we investigated a possible role in cognitive behaviors of PC catecholamines, by mapping and testing functional roles of Th positive PCs in mice. Comprehensive mapping analyses revealed a distinct population of Th expressing PCs primarily in the posterior and lateral regions of the cerebellum (comprising about 18% of all PCs). To identify the role of PC catecholamines, we selectively knocked out Th in PCs using a conditional knockout approach, by crossing a Purkinje cell-selective Cre recombinase line, *Pcp2-Cre*, with a floxed tyrosine hydroxylase mouse line *(Th^lox/lox^)* to produce *Pcp2-Cre;Th^lox/lox^* mice. This manipulation resulted in approximately 50% reduction of Th protein expression in the cerebellar cortex and lateral cerebellar nucleus, but no reduction of Th in the locus coeruleus, which is known to innervate the cerebellum in mice. *Pcp2-Cre;Th^lox/lox^* mice showed impairments in behavioral flexibility, response inhibition, social recognition memory, and associative fear learning relative to littermate controls, but no deficits in gross motor, sensory, instrumental learning, or sensorimotor gating functions. Catecholamines derived from specific populations of PCs appear to support cognitive functions, and their spatial distribution in the cerebellum suggests that they may underlie patterns of activation seen in human studies on the cerebellar role in cognitive function.

## Introduction

Mechanistically, a role for the cerebellum in cognition has been primarily understood from an anatomical perspective, with several studies focusing on how specific regions of the cerebellum have connectivity with other brain regions that are more traditionally associated with cognitive function, such as the ventral tegmental area (VTA) or prefrontal cortex (PFC; Kelly and Strick, 2003; Rogers et al., 2011; Watson et al., 2014; Parker et al., 2017; Locke et al., 2018; Carta et al., 2019). However, neurotransmitter systems classically known for their involvement in cognitive functions are abundant within the cerebellum. For example, dopamine (DA), norepinephrine (NE) and their metabolites have been observed in the cerebellar vermis, hemispheres, and each of the cerebellar nuclei in rodents (Bloom et al., 1971; Versteeg et al., 1976; Freedman, 1977; Freedman et al., 1977; Panagopoulos et al., 1991). DA and NE are neuromodulatory neurotransmitters that are well known for facilitating learning of associations of salient cues with specific outcomes and modulating cognitive processes guiding approach and avoidance behaviors (Schultz, 2013; Aston-Jones and Waterhouse, 2016). DA is found at a similar concentration in the whole cerebellum as it is in the hippocampus in mice (Laatikainen et al., 2013). Cerebellar nuclei have higher concentrations of norepinephrine and DA than the cerebellar cortex in the rat, as well as similar levels of these catecholamines relative to the frontal cortex (Versteeg et al., 1976). Furthermore, there is a rich literature on the role of NE, supplied extrinsically from the locus coeruleus (LC), in modulating classical cerebellar functions (Paredes et al., 2009). NE is necessary for cerebellar-dependent motor learning (Watson and McElligott, 1983, 1984), modulates plasticity of the vestibulo-ocular reflex (McElligott and Freedman, 1988), and is important for multiple phases of cerebellar-dependent classical conditioning of eyeblink responses, including acquisition, consolidation and extinction (McCormick and Thompson, 1982; Winsky and Harvey, 1992; Paredes et al., 2009).

These observations are important in the context of cerebellar cognitive function: first, subpopulations of Purkinje cells identified express tyrosine hydroxylase (Th), the rate-limiting enzyme in the synthesis of catecholamines (Hess and Wilson, 1991; Takada et al., 1993; Fujii et al., 1994; Abbott et al., 1996), as well as DA D3 receptors (Levant and DeSouza, 1993; Barik and de Beaurepaire, 1996, 2005), DA transporter and the vesicular monoamine transporter 2 and are thus likely to release DA (Kim et al., 2009). Second, TH expression in a subset of Purkinje cells is also seen during development in humans (Fujii et al., 1994). Third, Th expression is linked to evolutionary volume expansions in cerebellum thought to underlie cognitive evolution in humans (Harrison and Montgomery, 2017). Fourth, in subjects with Alzheimer’s Disease, an illness defined by its deficits in cognitive function, post-mortem catecholamine levels, Th expression, and adrenergic receptor expression in the cerebellum are strongly correlated with neuropsychiatric symptoms, more so, in fact than they are in frontal and temporal cortex (Russo-Neustadt and Cotman, 1997; Russo-Neustadt et al., 1998; Vermeiren et al., 2014a,b). However, the roles and sources of catecholamines in the cerebellum are not well understood, and specifically, the functional role of PC-derived catecholamines is unknown.

We hypothesized that Th expression in Purkinje cells could be important for the cognitive functions of the cerebellum. To test this hypothesis, we generated a line of mice in which Th was genetically deleted specifically in PCs and performed behavioral analyses in these mice to interrogate cognitive functions of the cerebellum. Comprehensive mapping of Th immunohistochemical and mRNA expression revealed a cerebellum-wide distribution of Th+ PCs with specific enrichment in posterior and lateral regions that have been implicated in cerebellar cognitive functions. PC-specific knockout of Th in mice resulted in specific impairments in behavioral flexibility, response inhibition, the rate of learning to discriminate between cues in an associative fear conditioning task, and social recognition memory, and notably, had no effect on gross motor function, or sensorimotor gating. These findings may imply that catecholamine-facilitated learning mechanisms, within the cerebellum, encode learning signals and are necessary for modulation of certain cognitive functions.

## Materials and Methods

### Mice

The University of Washington Institutional Animal Care and Use Committee approved all experimental protocols (4249-01). Heterozygous transgenic mice with Cre recombinase driven by the *Pcp2*-promoter *B6.129-Tg(Pcp2-Cre)2Mpin/J line* (Zhang et al., 2004) were crossed with *Th^lox/lox^* mice (previously described in Jackson et al., 2012) to produce* Pcp2-Cre;Th^lox/lox^* mice, which were genotyped by PCR. Littermates without Pcp2-Cre or those expressing only one or no *Th^lox^* allele (*Th^lox/+^ and Th^+/+^*) were used as controls. Only male mice were used for behavioral experiments in this study. Three to Five-month-old mice were given *ad libitum* food and water except during food restriction to 85% of their *ad libitum* body weight, while housed on a 12:12 light:dark cycle. For immunohistochemistry, adult C57/BL6 mice purchased from JAX were used under protocols approved by the Johns Hopkins University Animal Care and Use Committee (MO16M464).

### Immunohistochemistry

For immunostaining, mice were deeply anesthetized with 2,2,2-tribromoethanol (also known as avertin, 0.5 mg/g) and then were transcardially perfused with PBS containing heparin (10 U/ml) followed by 4% paraformaldehyde (PFA) in PBS. Brains were dissected out from the skull, postfixed overnight in 4% PFA in PBS and cryoprotected in 30% sucrose PBS. Those brains were then embedded in gelatin to prevent separation of the cerebellum from the brainstem. The gelatin blocks were hardened on ice and were then trimmed and fixed overnight with 4% PFA in 30% sucrose PFA. Coronal or sagittal serial sections were cut at a thickness of 40 μm with a freezing microtome. After rinsed with PBS containing 0.15% Triton-X (PBST), the sections were stored in PBS containing 0.1% sodium azide at 4°C until use.

Immunostaining was performed with Nickel-enhanced 3,3′-diaminobenzidine (DAB) visualization or fluorescent visualization. For the DAB processing, before antibody incubation, sections were treated three times with 1.0% hydrogen peroxide and 1.0% sodium azide dissolved in distilled water to quench intrinsic peroxidase activity. For both DAB and fluorescent visualization, sections were then rinsed with PBST and incubated Three overnights on a shaker at 4°C with the primary antibody, rabbit anti-tyrosine hydroxylase (1:300, AB152, Millipore, Kankakee, IL, USA), sheep anti-tyrosine hydroxylase (1:100, P60101, Pel Freez), and/or rabbit anti-Aldolase C (1:1,000, Dr. Izumi Sugihara). After being rinsed five times with PBST for more than an hour in total, sections were incubated overnight at 4°C with donkey secondary antibody conjugated with biotin (1:500, Jackson ImmunoResearch Laboratories Inc., West Grove, PA, USA), AlexaFluor 488, or AlexaFluor 594 (1:400 for fluorophores, Jackson ImmunoResearch Laboratories Inc., West Grove, PA, USA), and 5% NDS in PBST. Fluorescently stained sections were then rinsed with PBS, mounted on a sliding glass, and coverslipped with ProLong Glass Antifade Mountant (P36980, Thermo Fisher Scientific, Waltham, MA, USA). Sections for DAB visualization were further processed as follows. After rinsing with PBST as in the previous step, sections were then incubated overnight at 4°C with the ABC-kit solution (PK-6100, Vector Labs) according to the manufacturer’s recommendations. Then, followed by five times of rinsing with tris-buffered saline, sections were preincubated with filtered DAB solution (DAB 0.4 mg/ml, ammonium chloride 4 mg/ml, D-glucose 4 mg/ml, nickel ammonium sulfate 2 mg/ml) at dark for 10 min. The DAB reaction was initiated by adding glucose oxidase ( 0.8 mg/ml as reaction concentration, G6125, Sigma). The reaction was continued for 20–40 min at dark until the black labeling reached the desired level, and sections were thoroughly rinsed with PBS, mounted on a sliding glass, and coverslipped with DPX mount (Sigma). Chemicals were purchased from Sigma. Images were taken with a CCD camera (ORCA-100, Hamamatsu) attached with a microscope (BX61, Olympus) or a confocal microscopy (FV1000, Olympus), or were acquired using a 10× objective on a Keyence slide microscope, or a Nikon Eclipse E600 upright microscope, and processed using NIH ImageJ software. Contrast and brightness were adjusted in Photoshop CS6 (Adobe).

### Serial Section Alignment Analysis

To demonstrate the distribution of the Th+ PCs in the vermis, we applied the serial section alignment analysis (Fujita et al., 2012, 2014). In each photomicrograph from a set of coronal cerebellar sections of the same animal, immunostained for TH, the Purkinje cell bodies including some fraction of their dendrites were clipped into strips in the entire mediolateral extent of the vermis. These clipped strips were aligned to each other so that the aligned order reflected the continuity of the cerebellar cortical layers that were folded to form lobules of the intact cerebellum. Some clipped strips were rotated and/or shifted so that they would show good alignment with neighboring strips. We performed this analysis with every three serial sections. This analysis could not be performed in the hemispheric regions where extrinsic Th+ fibers innervating PCs and molecular layers were too dense to effectively identity Th+ PC cell bodies and dendrites.

### Verification of Th Knockout With Western Blot

Western blots of the lateral cerebellar nucleus, locus coeruleus, or parafloccular tissue isolated by hole punch of a section of cerebellum after the termination of the experiment were performed after tissue homogenization and protein extraction with 1:10,000 Millipore Rabbit anti-Th antibody AB152 stain, quantified after normalization to Millipore mouse anti-β-actin antibody stain, AB1501, concentration = 1:8,000.

### Behavioral Analysis

Male animals were run on our behavioral pipeline in a fixed temporal order. The pipeline for this study consisted of social interactions (1 day), rotarod (4 days), startle curve measurement and prepulse inhibition of the acoustic startle reflex (PPI; 1 day), Fixed Ratio 1 (FR1) lever training (5 days), delayed alternation and reversal (DA; 10 days), elevated plus maze (1 day), and fear conditioning (3 days), followed by another round of elevated plus maze (1 day), for a total of approximately 1 month (26 days) of behavioral assays. Additional male animals were generated to test on the same pipeline, but instead of delayed alternation, they were run on the differential reinforcement of low rate responses (DRL task) for 4 days, for a total of approximately 3 weeks (20 days) of behavioral assays.

### Fear Conditioning Behavior

In order to test attention to salient sensory stimuli, we used a discriminative delayed cued fear conditioning paradigm in which the subject was exposed to an auditory cue (a specific tone, CS+) predictive of a low amplitude shock (0.3 mA, 0.5 s) and an auditory cue that was not predictive of the shock (CS−). This test required the subject to discriminate between cues in the context of a predicted shock; learning was measured by the amount of freezing after playing CS+ or CS− during the probe trial. This test typically does not result in generalized fear, and likely requires more attention than tests with higher-amplitude shocks (Sanford et al., 2017). Conditioning and test sessions were performed in a standard operant chamber (Med Associates Inc.) equipped with a tone generator and house light. Baseline responses to two distinct auditory stimuli (10 kHz pulsatile tone and a 20 kHz continuous tone, each 10 s in duration) were established by three interleaved presentations of the cues. Mice were then conditioned to CS+ presentations (10 s auditory cue) co-terminating with the US and CS− presentations (distinct 10 s auditory cue that did not co-terminate with a US) on two consecutive days. Daily sessions that were repeated over two consecutive days consisted of 10 presentations of the CS+ co-terminating with a 0.3 mA foot shock alternating pseudo-randomly with 10 presentations of the CS− on a 60 s intertrial interval (ITI). The assignment of the tones as the CS+ and CS− was counterbalanced across groups. Twenty-four hours after conditioning, on two consecutive days, mice were probed for discriminative threat responding by monitoring freezing in response to three interleaved presentations of the CS+ and CS−, which were delivered at a 60 s interval in the absence of the US. Test sessions were conducted in a different context from the conditioning, consisting of solid white walls and flat white floor with acetic acid olfactory cues. All sessions were analyzed with Ethovision (Noldus) tracking software after video recording.

### Elevated Plus Maze

Mouse entries into, and time spent in all areas of an elevated plus-maze (Med Associates), was monitored for 10 min 1–3 days before discriminative delayed cued fear conditioning and 1–3 days following conditioning using a video acquisition system. Data were analyzed using Ethovision tracking software (Noldus).

### Prepulse Inhibition of the Acoustic Startle Reflex

Prepulse inhibition was performed in sound-attenuated chambers (San Diego Instruments, San Diego, CA, USA). To measure prepulse inhibition, mice were given five trials of 120 dB startle pulse-alone, followed by 50 trials which pseudorandomly alternated between 120 dB pulse-alone, one of three prepulse intensities, or null (no startle), with a variable ITI. Prepulse trials consisted of 20 ms duration prepulse at the indicated intensity occurring 100 ms before the 40 ms 120 dB startle pulse. Acoustic startle curves were measured similarly 1 day before measurement of PPI, but without prepulse tones, at each of six different amplitudes of a startle tone.

### Rotarod

Mice were placed on a rotating rod apparatus (Columbus Instruments, Columbus, OH, USA) with increasing speed (4–40 rpm over 300 s; performed on a 3 cm diameter rod spindle, with a 44.5 cm fall height), and the latency to fall was recorded. This procedure required three sessions a day for 5 days for each animal.

### Operant Behaviors

Before DRL or DA, mice were calorie-restricted to 85% of their baseline weight and lever trained [fixed ratio 1 (FR1): one press for one pellet] in Med Associates operant chambers. In the delayed alternation task, the subject chose one of two levers, levers retracted, and then there was a delay of 2 s; FR1 training took 4 days, and delayed alternation took 6 days of training for a 2 s delay duration. The reversal of the alternation was then performed for 4 days, with a delay of 8 s. In the reversal, a subject chose one of 2 levers, levers retracted, and then, instead of pressing the opposite lever in the original alternation, they are required to press the same lever after the delay.

### Instrumental Conditioning

Conditioning was performed in sound-attenuated chambers (Med Associates, Georgia, VT, USA). To test simple reward learning and motivation, we used an established appetitive simple instrumental conditioning task (Gore and Zweifel, 2013; Locke et al., 2018). Calorie restricted (to 85% of body weight) mice were monitored for learning in a fixed-ratio 1 (FR1) reward for one lever press results in the delivery of a food pellet. Four days of instrumental conditioning were performed in male mice. The session continued until 50 trials were completed or 2 h had elapsed.

### Differential Reinforcement of Low Rate Responding

To gauge impulsivity, we used the DRL operant task paradigm as previously described (Nautiyal et al., 2015), with minor alterations. Mice began training on the DRL-10 paradigm the day after they completed 5 days of lever training on an FR1 schedule. In the DRL-10, responses before 10 s have elapsed after the last press result in a reset, while presses that are preceded by at least 10 s are rewarded. Mice were trained daily for 4 days at the 10-s interval. All sessions lasted 1 h, and no limit was placed on the number of rewards the animal could receive (but the physical limit would be 360 rewards). When catecholamines are depleted in the PFC, performance on this task is decreased in rats (Sokolowski and Salamone, 1994). Experimental and control mice on both operant behaviors were compared using a Two-way RM ANOVA (group × day).

### Delayed Alternation and Reversal

To gauge working memory, we used an operant version of delayed alternation paradigm as previously described with minor alterations (Rossi et al., 2012; Locke et al., 2018). Mice began training on the delayed alternation paradigm the day after they completed 4 days of lever training on an FR1 schedule. In this paradigm, mice are first presented with a free choice of pressing one of two levers. After choosing which lever to press, both levers are retracted, and a time interval of 2 s ensues. After this delay, the levers are then presented, and the mouse must learn to press the opposite lever than was originally pressed. There is a 20 s inter-trial interval, and the process is repeated. Mice were trained daily for 1 week on a 2 s delay interval. The percentage of correct alternations and pellets rewarded is measured. Experimental and control mice on both operant behaviors were compared using a Two-way RM ANOVA (group × day).

### Three Chamber Social Assay

Social interactions in male mice were assessed using a 3-chambered test as previously described (Silverman et al., 2010; Locke et al., 2018). Animal activity was recorded on video and analyzed using Ethovision Software (Noldus, Leesburg, VA, USA). The test mouse was placed in the center chamber at the beginning of each session. A cylindrical wire cage was used as an inanimate object or the cage housing a stranger mouse. The mouse then freely explored each chamber for 10 m. In the second 10-min session, an age- and gender-matched C57BL/6J mouse that had never been exposed to the subject was placed in one of the two-wire cages. Then, the test mouse freely explored the chamber again for 10 min. The test mouse was removed and, in the last 10-min session, a second age- and gender-matched novel stranger mouse that had never been exposed to the test mouse was placed in one cage, which previously served as the empty cage. Thus, the test mouse had the choice between a mouse that was already familiar and a new stranger mouse. The test mouse was again allowed to freely explore the chamber for 10 min. Time spent in each chamber was measured.

### Statistical Analyses

Data were analyzed using Excel (Microsoft, Redmond, WA, USA) and Prism software (GraphPad, San Diego, CA, USA), using ANOVA (with repeated measures and appropriate *post hoc* analyses when indicated), or Student’s *t*-test as indicated. *P* < 0.05 was considered significant. No randomization was used to assign experimental groups, but groups were assigned without bias. One animal was removed from the delayed alternation due to the failure of the lever apparatus. Behavioral data were acquired and analyzed in an unbiased way by an investigator without knowledge of the experimental groups.

## Results

### Mapping Tyrosine Hydroxylase Positive Purkinje Cells in the Mouse Cerebellum

**Figure 1:**
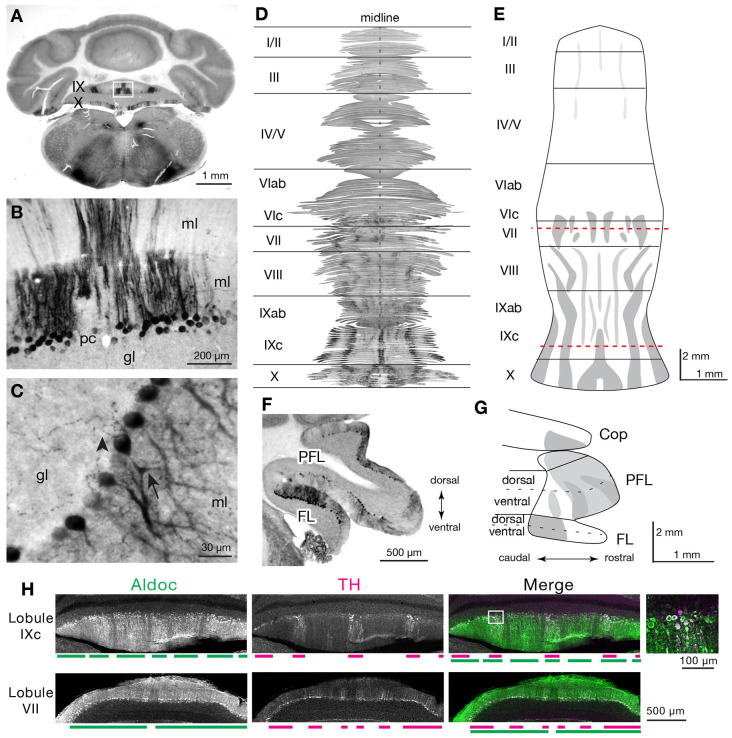
Immunostaining for Th reveals the striped distribution of the Th+ Purkinje cells (PCs) in the cerebellar cortex of the posterior vermis and parafloccular/floccular complex. **(A)** Immunostaining for Th reveals physiological Th expression in the cerebellum. Expression in lobules IX and X are shown in the coronal section. **(B)** Robust immunoreactivity for Th is identified in a subset of Purkinje cells that are arranged in parasagittal stripes. Cell bodies and dendrites of these PCs were labeled. ml, molecular layer; pc, Purkinje cell layer; gl, granule cell layer. **(C)** High magnification of Th+ PCs demonstrates Th+ puncta (arrowhead) that are in close apposition to PCs. Purkinje cell bodies and apical dendrites (Arrow) again show intense immunoreactivity for Th. ml, molecular layer; pc, Purkinje cell layer; gl, granule cell layer. **(D)** Serial section alignment analysis for the entire vermal cortex to visualize stripes of Th+ PCs. The result demonstrates an unfolded flat map of the vermal cortex reconstructed from the serial cerebellar sections immunostained for Th (see “Materials and Methods” section). **(E)** Schematic representation of the striped distribution of the Th+ vermal PCs. Drawn from the analysis in panel **(D)**. Red dotted lines indicate the approximate location of the images in panel **(H)**. **(F)** Th-immunoreactive PCs identified in the parafloccular/floccular (PFL/FL) complex. **(G)** Schematic representation of the distribution of the Th+ parafloccular/floccular PCs. **(H)** Double immunostaining for Th and Aldoc of cerebellar sections. Colabeling of Th (magenta) and Aldoc (green) is revealed in a subset of Aldoc+ Purkinje cells. Images of lobules IXc or VII, at levels indicated in **(B)**, are shown. Below each panel is magenta and green bars that represent striped expression patterns of Th and Aldoc, respectively. Inset shows a higher magnification image of PCs, at an area marked with a white rectangle, that is colabeled with Th and Aldoc.

Several reports have noted the presence of Th expression in a subset of Purkinje cells across species (Hess and Wilson, 1991; Takada et al., 1993; Fujii et al., 1994; Abbott et al., 1996). Here, we comprehensively map cerebellar Th expression and show that it is enriched in cerebellar regions implicated in cognitive function. To elucidate the cellular and subcellular expression of Th in PCs, we assessed the expression of Th in immunostained cerebellar sections (Figures 1A–G). We confirmed strong Th+ PC expression in the cerebellar lobules IX and X (Figures 1A,B), paraflocculus (Figures 1F,G), and flocculus (Figures 1F,G). Several PCs exhibit high expression of Th in cell bodies and apical dendrites extending into the molecular layer (Figures 1B,C arrow, Figure 1F). Th+ immunopositive puncta were observed in the granular cell layer and could reflect expression in recurrent collaterals of the PC axons (Figure 1C, arrowhead).

To comprehensively assess the distribution of Th+ PCs, we performed a cerebellum-wide serial section analysis of Th expression in PCs in the mouse (Figure 1D). We then used this data to make a map of Th expression in the vermis and parafloccular/floccular complex (Figures 1D–G). Th+ Purkinje cells were arranged in several longitudinal stripes in the central (VIc–VII) and posterior vermis (VIII–XI), and nodulus (X), with the strongest expression in the lobule IXc. PCs in the anterior vermis were barely stained for Th. The paraflocculus contained Th+ PCs mainly in its rostral part, and the Th+ PCs in the flocculus were clustered in its caudal part (Figures 1F,G).

Each of the parasagittally arranged band of PCs is known to have distinct circuit connections with the downstream cerebellar nuclei and upstream climbing fiber projections from the inferior olive (Oscarsson, 1979; Voogd and Glickstein, 1998). These bands of PCs exhibit alternating expression of Aldoc (zebrin II) and the correspondence between Aldoc stripes, circuit connections, and their functions have been well studied (Itō, 1984; Apps and Hawkes, 2009; Apps et al., 2018). To assess the correspondence between Th stripes and Aldoc stripes, we performed double immunostaining for these markers on the cerebellar sections. The resultant labeling demonstrated that Th expression was detected only in the Aldoc-immunopositive PCs. In the vermal regions, labeling for Th overlapped with subsets of Aldoc-immunopositive PC (Figure 1H). Although it was challenging to visualize weak TH expression of the Crus I PCs with reliable immunofluorescent staining intensity, it would be reasonable to assume that the TH+ PCs in the Crus I and PFL overlap with Aldoc expression since these hemispheric regions are known to all express Aldoc (Apps and Hawkes, 2009; Fujita et al., 2014).

**Figure 2:**
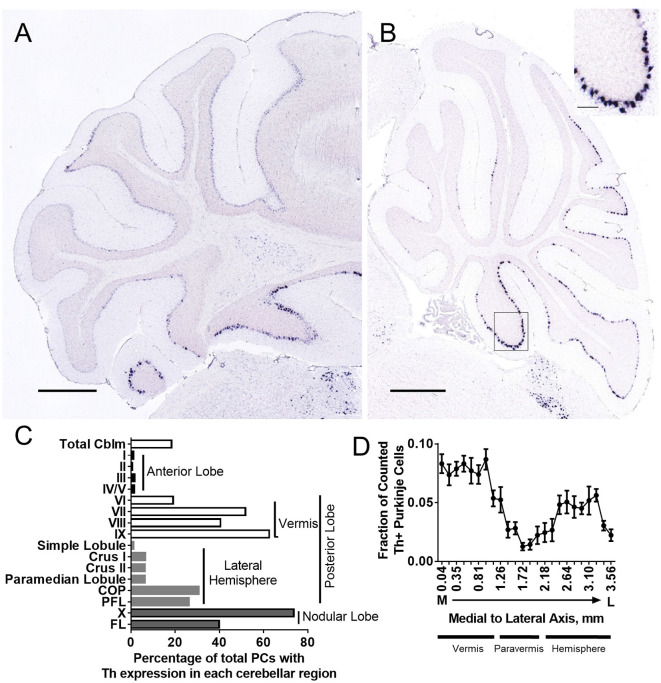
*Th+* Purkinje cell counting with *in situ* hybridization (ISH). *Th*+ Purkinje cells (dark blue) populate multiple regions of the cerebellum. *Th* mRNA was measured in six brains from the Allen Brain Atlas (Table 1). **(A)** Coronal section from Allen Mouse Brain Atlas (2004). Image credit: Allen Institute (Experiment 1056; Lein et al., 2007). **(B)** Sagittal section from Allen Mouse Brain Atlas (2004). Image credit: Allen Institute (Experiment 1058; Lein et al., 2007). Scale bar = 652 microns. *N* = 6 brains. Inset, higher magnification from area in square. Inset scale bar = 100 microns. **(C)** Estimated percentage of Purkinje cells with *Th*+ expression in each cerebellar cortical region. **(D)** A fraction of counted *Th*+ Purkinje cells in sagittal sections along medial to the lateral axis. Error bars are SEM.

To further confirm Th expression in mouse cerebellum, we analyzed six brains from the Allen Brain Atlas (Lein et al., 2007) in which *in situ* hybridization was performed for the *Th* mRNA transcript. Sagittal and coronal sections show distinct *Th* mRNA expression in PCs (Figures 2A,B). We also analyzed four brains from the Allen Mouse Brain Atlas with *in situ* hybridization for genes (*Pcp2, Syt7, Gng13, Slc32a1*) enriched in all PCs to estimate total numbers of PCs in mouse brain. We then estimated the percentage of PCs that were Th+ in total cerebellum (approximately 18%) and each cerebellar subregion and found a reliable and specific pattern of expression (Figure 2C). We also plotted this as a fraction of counted Th+ PCs (Supplementary Figure S1). *Th* mRNA was prominent in posterior lobules VI–X, with high expression in the vermis, low expression in the paravermis, and medium expression in the lateral hemispheres (Figures 2C,D).

### Generation of a Purkinje Cell-Specific Knockout of Tyrosine Hydroxylase

**Figure 3:**
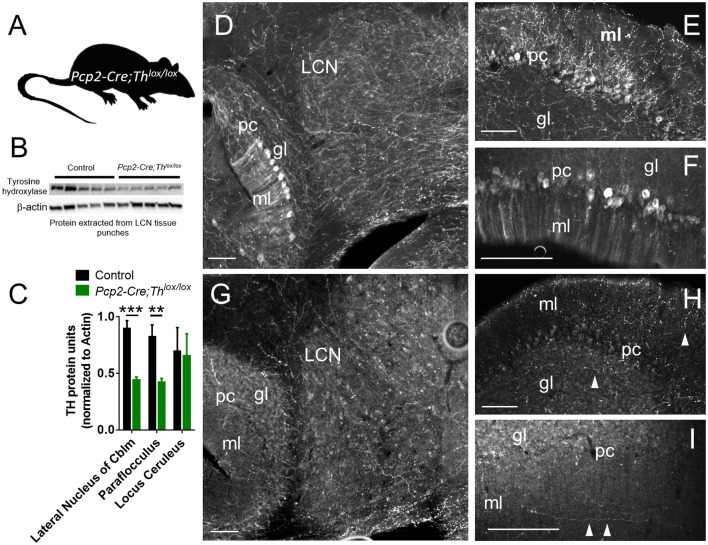
Generation and confirmation of mouse with conditional deletion of Th in Purkinje Cells. **(A)** Schematic for Purkinje Cell-specific knockout of Th: *Pcp2-Cre* mice crossed with *Th^lox/lox^* mice to generate *Pcp2-Cre;Th^lox/lox^* mice. **(B)** Western Blot from LCN with five representative controls on the left and five *Pcp2-Cre;Th^lox/lox^* mice on the right. The top band is Th, the β-actin band is on the bottom. **(C)** Quantification of Western Blots, from LCN, paraflocculus, and locus coeruleus. Error bars are SEM. Each Th band normalized to β-actin. ***p < 0.0003, one-tailed *t*-test, *t*_(8)_ = 6.03. **p < 0.0065, one-tailed *t*-test, *t*_(8)_ = 3.69. For LC comparison, *p* = 0.9. **(D–I)** Staining of Th in the red color channel in LCN, paraflocculus, and vermis (Lobule IX) of control **(D–F)** and *Pcp2-Cre;Th^lox/lox^* mice **(G–I)**. Labels: ml = molecular layer, pc = Purkinje cell layer, gl = granule cell layer. Scale bars = 100 microns. The manipulation resulted in a significant reduction in the Th+ fibers innervating the LCN **(D** vs. **G)**, and complete deletion of the Th signals in the PCs **[D** vs. **G**, **E** vs. **H**, for the paraflocculus, **F** vs. **I** for the posterior vermis (Lobule IX)**]**, though some Th fibers (white arrowheads) are still present in the ml and gl layers in *Pcp2-Cre;Th^lox/lox^* mice, likely representing extrinsic sources of Th.

To specifically assess the effects of catecholamines derived from Th+ PCs, without confounding influences of catecholamines derived from LC, we generated mice with conditional deletion of *Th* expression in PCs. Targeted *Th^lox/lox^* mice in a C57BL/6J background strain (Jackson et al., 2012) were crossed with mice expressing a *Pcp2* gene promoter-driven Cre recombinase (Cre) transgene (*Pcp2-Cre*) [*B6.129-Tg(Pcp2-Cre)2Mpin/J* line (Zhang et al., 2004)] in a C57BL/6J background to generate a double mutant, Purkinje cell neuron-specific knockout of *Th* (*Pcp2-Cre;Th^lox/lox^* mice) (Figure 3A). *Pcp2-Cre;Th^lox/lox^* mice were verified by the Western blot of LCN tissue isolated by the hole punch of a section of the cerebellum (Figures 3B,C). Littermates without Pcp2-Cre or those expressing only one or no *Th ^lox^* allele (*Th^lox/+^ and Th^+/+^*) were used as controls. Tyrosine hydroxylase protein was reduced by 50% in the lateral cerebellar nucleus (LCN: Figure 3C). We additionally confirmed Purkinje cell knockout of Th with immunohistochemical staining for Th in coronal sections of LCN, paraflocculus, and cerebellar cortex in control mice (Figures 3D–F) and *Pcp2-Cre;Th^lox/lox^* mice (Figures 3G–I). Additionally, we verified *Th* knockout with Western blot from hole punches of the cerebellar cortex from paraflocculus and flocculus, as well as the LC (Figure 3C and Supplementary Figure S2A). Th protein expression was significantly decreased by approximately 50% in paraflocculus and flocculus, yet no difference was seen between groups in Th protein expression in LC, indicating the specificity of this manipulation to PCs, and that many of the Th+ fibers in cerebellum originate from PCs. Th+ fibers were present in the molecular layer of the cerebellar cortex, even after PC-specific deletion of Th, which suggests that these Th+ fibers are from a different source (likely LC) than PCs (Figures 3F,I).

### PC-Derived Catecholamines Modulate Pavlovian Fear Learning

**Figure 4:**
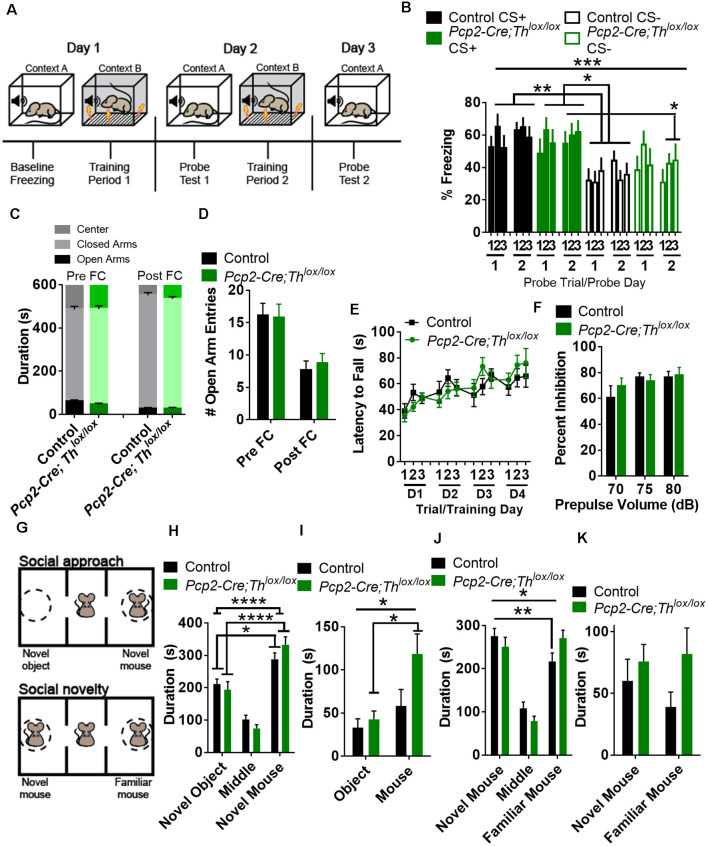
Conditional knockout of *Th* in all Purkinje cell fibers results in altered fear conditioning and social recognition memory, but not anxiety, sensorimotor gating, or gross motor deficits. **(A)** Schematic for fear conditioning experiment. **(B)** Freezing after fear conditioning of *Pcp2-Cre;Th^lox/lox^* mice (Green, *n* = 10) and littermate controls (Black, *n* = 18) on a fear discrimination task with a low-amplitude shock (0.3 mA) in both groups showed an increased association of CS+ to shock on the probe. There was an effect of genotype, *F*_(3,52)_ = 6.59, *p* = 0.0007, but not an effect of training *F*_(5,260)_ = 1.37, *p* = 0.236, or an effect of interaction between genotype and training *F*_(15,260)_ = 0.96, *p* = 0.498, Two-way rmANOVA. On *post hoc* analyses (Tukey’s multiple comparisons test) Control mice discriminated between CS+ and CS− over the entire period (**p < 0.01), while *Pcp2-Cre;Th^lox/lox^* mice did not. Both groups discriminated between CS+ and CS− after 2 days of conditioning, *p < 0.05. Error bars are SEM. **(C)** Time spent in the center area, open and closed arms before (Pre FC), and after (Post FC) fear conditioning. *Pcp2-Cre;Th^lox/lox^* mice (Green, *n* = 10) and littermate controls (Black, *n* = 18) mice showed no differences in time spent in any region before or after fear conditioning. Error bars are SEM. **(D)** Number of entries into open arms before (Pre FC) and after (Post FC) fear conditioning. *Pcp2-Cre;Th^lox/lox^* mice (Green, *n* = 10) and littermate controls (Black, *n* = 18) mice showed no differences in time spent in open arms before or after fear conditioning. Error bars are SEM. **(E)** Performance of control (black, *n* = 9) and *Pcp2-Cre;Th^lox/lox^* mice (green, *n* = 6) on accelerating rotarod over 4 days of training. No difference observed between groups or the effect of group interaction with training. Error bars are SEM. **(F)** Prepulse inhibition of the acoustic startle reflex was not significantly different between groups; *Pcp2-Cre;Th^lox/lox^* mice (Green, *n* = 10), controls (Black, *n* = 10). No difference observed between groups or the effect of interaction between group and prepulse volume. Error bars are SEM. **(G)** Schematic of social interaction assays. **(H)** Social approach as measured by time spent in the arena with a novel mouse or novel object. Factor for time spent in zone(*F*_(2,78)_ = 71.53, *p* < 0.0001) was significant, but interaction (genotype group × zone, *F*_(2,78)_ = 2.22, *p = 0.115) and genotype (*F*_(1,78)_ < 0.0001, *p* = 0.996) were not. On *post hoc* analysis, both controls and *Pcp2-Cre;Th^lox/lox^* had preferences for novel animal over novel objects (Black, *n* = 11, *p < 0.05), *Pcp2-Cre;Th^lox/lox^* mice (Green, *n* = 7, ****p < 0.0001), Two-way rmANOVA, Sidak’s multiple comparison’s test. Error bars are SEM. **(I)** Social approach as measured by time spent in interaction zones with a novel mouse or novel object. Factors for time spent in interaction zone (*F*_(1,16)_ = 20.63, ***p < 0.001) and interaction (genotype group × zone, *F*_(1,16)_ = 5.2, *p < 0.05) were significant, but genotype (*F*_(1,16)_ = 2.79, *p < 0.114) was not. On *post hoc* analysis, only *Pcp2-Cre;Th^lox/lox^* had preferences for novel animal interaction zone over the novel object (Green, *n* = 7, ***p < 0.001), Two-way rmANOVA, Sidak’s multiple comparison’s tests. Error bars are SEM. **(J)** Social preference as measured by time spent in the arena with a novel or familiar mouse. Factors for interaction (genotype group × zone, *F*_(2,32)_ = 3.87, *p < 0.05) and zone (*F*_(2,32)_ = 25.33, *p* < 0.0001) were significant, as well as for time spent (preference for novel mouse over a familiar mouse) in control mice (Black, *n* = 11, **p < 0.01), but not *Pcp2-Cre;Th^lox/lox^* mice (Green, *n* = 7) on *post hoc* analysis. Two-way rmANOVA, Sidak’s multiple comparison test. Error bars are SEM. **(K)** Time spent in interaction zones in the social novelty phase of the test. No significant difference in the factors of time, genotype or interaction was found between controls and *Pcp2-Cre;Th^lox/lox^* mice in this comparison. Error bars are SEM.

As the cerebellum is known to process signals predicting perceptual cues (O’Reilly et al., 2008), we hypothesized that *Pcp2-Cre;Th^lox/lox^* mice would exhibit decreased discrimination between predictive and non-predictive cues. To test attention to salient sensory stimuli, we used a fear conditioning paradigm in which the subject is exposed to two types of auditory cues: a specific tone (CS+), predictive of a low amplitude foot shock (0.3 mA, 0.5 s) and a tone cue of different frequency that is not predictive of the shock (CS−; Figure 4A). This test requires the subject to learn to a cue that predicts a shock and discriminate between cues to freeze in the context of a predicted shock; learning is measured by the amount of freezing after playing CS+ or CS− during the probe trial. Over the entire testing period, controls learned to differentiate CS+ from CS− (Figure 4B, Supplementary Figure S2B). *Pcp2-Cre;Th^lox/lox^* mice learned to associate CS+ with shock, and no differences were found between Controls and *Pcp2-Cre;Th^lox/lox^* mice in freezing for CS+. When we compared *Pcp2-Cre;Th^lox/lox^* mice CS+ to Control CS−, there was significantly more freezing in the *Pcp2-Cre;Th^lox/lox^* mice CS+ group (Figure 4B). However, over the entire testing period, there was no significant difference between freezing to CS+ or CS− in the *Pcp2-Cre;Th^lox/lox^* mice, meaning that *Pcp2-Cre;Th^lox/lox^* mice were unable to discriminate tones that did or did not predict shock. Over the entire testing period, there was also no difference between Control CS+ and *Pcp2-Cre;Th^lox/lox^* CS−. On *post hoc* analysis, *Pcp2-Cre;Th^lox/lox^* did show a significant difference in freezing to CS+ compared to CS−, but only after 2 days of training. This difference, the slower rate in sensory discrimination learning, did not appear to be related to anxiety, as *Pcp2-Cre;Th^lox/lox^* mice showed no differences in the exploration of an elevated plus-maze, as measured by time in each region of the maze or number of entries into open arms when compared to controls (Figures 4C,D). This difference also does not appear to be a result of a gross motor deficit, as *Pcp2-Cre; Th^lox/lox^* did not have changes in performance on the accelerating rotarod (Figure 4E, Supplementary Figure S2E) which is a cerebellar dependent task.

We also assessed prepulse inhibition of the acoustic startle reflex (PPI) as a standard test for sensorimotor gating. PPI is thought to engage pre-attention systems, may influence freezing to a cue, requires intact auditory function, and is modulated by the cerebellum (Takeuchi et al., 2001; Locke et al., 2018). *Pcp2-Cre;Th^lox/lox^* mice did not show any differences in PPI compared with controls (Figure 4F; Supplementary Figure S2F).

### PC-Derived Catecholamines Influence Social Recognition Memory

Decreased social cognition is associated both with multiple mental illnesses and with neurodegenerative diseases associated with changes in the cerebellar function (Bauman and Kemper, 2005; Penn, 2006; Van Overwalle et al., 2014; Schmahmann, 2019; Schmahmann et al., 2019). Preference for social novelty is influenced by LCN cells expressing the DA D1 receptor (Locke et al., 2018). To determine whether social recognition memory requires Th+ PCs, we tested social preference with the three-chamber assay (Figure 4G; Supplementary Figure S2G, Silverman et al., 2010; Locke et al., 2018). When we performed the social approach phase of the assay, we found that both controls and *Pcp2-Cre;Th^lox/lox^* mice preferred to spend time in the novel mouse chamber vs. the novel object chamber (Figure 4H; Supplementary Figure S2G). *Pcp2-Cre;Th^lox/lox^* mice spent significantly more time in the interaction zone with the novel mouse than the novel object, whereas controls did not have a preference for either interaction zone in the social approach phase (Figure 4I). After the social approach phase of this assay, we replaced the object with a novel mouse, establishing a familiar mouse in one chamber and an unfamiliar mouse in the second chamber. Control mice spent more time in the chamber with the novel mouse, while *Pcp2-Cre;Th^lox/lox^* mice failed to discriminate between novel and unfamiliar mice in this task (Figure 4J, Supplementary Figure S2H). Neither group had a preference for either interaction zone in the social novelty phase of this task (Figure 4K).

### PC-Derived Catecholamines Modulate Behavioral Flexibility Involving Working Memory

**Figure 5:**
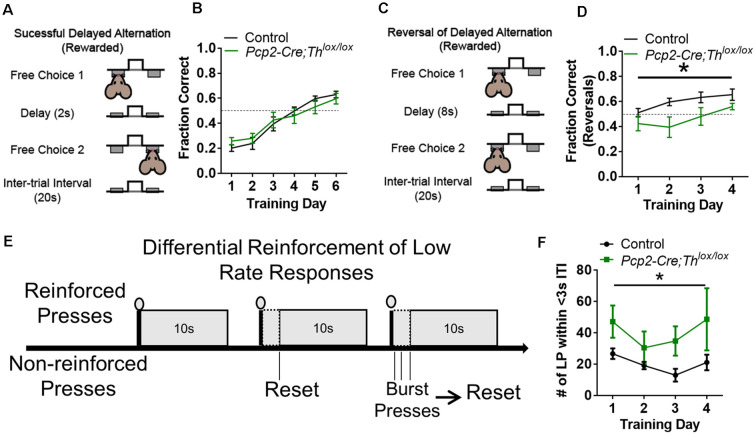
Conditional knockout of *Th* in all Purkinje cells results in altered behavioral flexibility and response inhibition, but not instrumental learning, or working memory. **(A)** Schematic of delayed alternation test for working memory, adapted from Beas et al. (2017). **(B)** Performance between *Pcp2-Cre;Th^lox/lox^* mice (Green, *n* = 8) and littermate controls (Black, *n* = 8) in delayed alternation with a 2-s delay. No difference observed between groups or the effect of interaction between group and time. Error bars are SEM. **(C)** Schematic of reversal of delayed alternation test for behavioral flexibility, adapted from Beas et al. (2017). **(D)** Performance of *Pcp2-Cre;Th^lox/lox^* mice (Green, *n* = 8) relative to littermate controls (Black, *n* = 8) in performance of delayed alternation reversal with 8-s delay. There was an effect of genotype group, *F*_(1,13)_ = 5.96, *p < 0.03 and training *F*_(3,39)_ = 6.68, *p < 0.001, but not for an effect between group × training *F*_(3,39)_ = 1.23, *p* = 0.313. Two-way rmANOVA. Error bars are SEM. **(E)** Schematic of differential reinforcement of low rate responses test for impulsive pressing, adapted from Nautiyal et al. (2015) and Locke et al. (2018). **(F)** Burst pressing performance (presses <3 s after last rewarded press, ITI = intertrial interval) of *Pcp2-Cre;Th^lox/lox^* mice (Green, *n* = 6) relative to littermate controls (black, *n* = 7) on DRL task over 4 days of training. There was an effect of group, *F*_(1,11)_ = 6.59, *p < 0.03. There was not an effect of training or the effect of interaction between group and training. Two-way rmANOVA. Error bars are SEM.

We hypothesized that the deletion of Th from PCs would result in decreased performance on a working memory task; instead, we found that this manipulation resulted in deficits in behavioral flexibility. Delayed alternation, a term for several similar tasks requiring working memory which can be evaluated in humans and animals, is deficient in neuropsychiatric disorders associated with prefrontal cortical catecholaminergic dysfunction including autism, Alzheimer’s disease, attention deficit disorder, schizophrenia, and frontal lobe disease (Freedman and Oscar-Berman, 1986a,b; Park and Holzman, 1992; Freedman, 1994; Arnsten and Li, 2005; Arnsten, 2006; Berridge et al., 2006; Loveland et al., 2008; Rossi et al., 2012). Performance on delayed alternation tasks, over longer intervals of delay, requires an intact PFC (Rossi et al., 2012) and LCN neurons expressing D1Rs (Locke et al., 2018). *Pcp2-Cre;Th^lox/lox^* mice did not show differences in learning in a delayed alternation task with a 2 s delay (Figures 5A,B, Supplementary Figure S2J). However, when we changed the task to a reversal of the rule (subjects had to press the same lever as the previous press) and increased the delay to 8 s (Figure 5C), then, *Pcp2-Cre;Th^lox/lox^* mice showed significantly decreased performance (Figures 5B–D, Supplementary Figure S2K). Since we altered both time and the rule, we cannot fully attribute this deficit to either working memory maintenance (time) or behavioral flexibility (change of the rule).

### PC-Derived Catecholamines Modulate Response Inhibition

We hypothesized that Th+ PCs would play a similar role as catecholaminergic modulation of response inhibition in prefrontal cortical circuits (reviewed in Arnsten and Li, 2005). To test this, we chose an operant task called differential reinforcement of low rate responding (DRL) which assesses the ability to inhibit responses until a specific time has elapsed (Figure 5E; Nautiyal et al., 2015). We assessed DRL learning by quantifying the number of lever presses within 3 s after the last rewarded press (which we refer to as “impulsive pressing”). *Pcp2-Cre;Th^lox/lox^* mice showed more impulsive pressing after the last press (or more failure to inhibit responses) than littermate controls over several weeks of training (Figure 5F, Supplementary Figure S2L). Deficits in performance on DRL were likely not due to motor deficits, as no differences in the ability to press a lever for a food reward were seen over 4 days of fixed-ratio 1 (FR1) lever training between groups (Supplementary Figure S2I).

## Discussion

In this study, we have confirmed and quantified the location and begun to characterize the role of Th+ PC-derived catecholamine signaling in the cerebellum in the mouse, with a focus on cognitive behaviors. Catecholamines provide a “learning signal” that acts to alter the excitability of cells receiving their inputs *via* their canonical action on G-protein coupled receptors and non-canonical action on β-arrestin (Harley, 2004; Urs et al., 2014; Schultz et al., 2017). Previous studies have shown that catecholaminergic signaling in the PFC, thalamus, amygdala, and striatum are important for top-down and bottom-up control of cognitive functions, particularly in explorative and exploitative cognitive strategies in approach and avoidance behaviors in novel contingencies or environments (Aston-Jones and Cohen, 2005; Yu and Dayan, 2005). Studies linking cerebellum to these behaviors have mostly focused on its connections with other brain regions, e.g., thalamus and VTA, but here, we show that catecholaminergic signaling, within a subset of PCs in the cerebellum, modulates Pavlovian fear conditioning, social recognition memory, working memory involving behavioral flexibility, and response inhibition. It is also notable that in this study, we found a specific pattern of Th+ PCs in different regions of the cerebellum, and particularly enriched in lobules VI-X of the vermis and Crus I and Crus II of the hemispheres. This pattern is remarkably similar to the spatial distribution of the cocaine- and amphetamine-regulated transcript peptide (CART) expression in a specific subset of climbing fibers arising from the inferior olive (Reeber and Sillitoe, 2011). CART has been shown to regulate memory, motivation, and fear in rodents (Chaki et al., 2003; Couceyro et al., 2005; Upadhya et al., 2011). Furthermore, we showed that the distribution of Th+ PCs was a subpopulation of the Aldoc+ PCs but not of the Aldoc- PCs that are thought to be primarily responsible for cerebellar motor functions (Voogd, 2014). Crus I and II, which are largely located by Aldoc+ PCs, exhibit evolutionary expansion in size in humans, when compared to other non-human primates (Chimps and Capuchin monkeys; Balsters et al., 2010; Luo et al., 2017). These neocerebellar regions also show activations during different cognitive, non-motor functions (Stoodley and Schmahmann, 2009, 2010; Stoodley et al., 2012; Schmahmann et al., 2019) and participate in known non-motor intrinsic connectivity networks (Buckner et al., 2011). To our knowledge, this is the first report of the behavioral consequences in cognitive functional domains of cerebellar catecholamines. This study shows that catecholamine signaling, intrinsic to the cerebellum, is yet another locus of catecholaminergic neuromodulation of cognitive functions. Given that cerebellar Th expression is linked to the evolutionary expansion of cerebellar volume thought to underlie cognitive functions (Harrison and Montgomery, 2017), it is interesting to speculate about why.

An important caveat for the interpretation of our behavioral findings is the possible effect of compensatory changes against conditional deletion of Th from PCs in our mouse model. We used the *B6.129-Tg(Pcp2-Cre)2Mpin/J* mouse, in which activity of the Cre recombinase in the PCs become detectable at postnatal day 6 (Zhang et al., 2004). Therefore, it is possible that the Th deletion from PCs in this study would start at P6, and that animal development after P6 would be affected. Particularly, developmental processes in the cerebellum including neurogenesis, axon guidance, dendritic development, synaptogenesis, and synapse refinement are still ongoing during this postnatal stage till atleast 4th postnatal week (Leto et al., 2016). Although Th function in the cerebellar development is an interesting research avenue, very little, if anything, is known about how deletion of Th affects later stages of neural development and morphology in general anywhere in the CNS.

Catecholamines have a wide variety of functions in the central nervous system ranging from the regulation of baseline activity, arousal, and locomotion to the modulation of fear and reward. Notably, there are several recent reports that the cerebellar cortex processes non-motor signals related to fear (Sacchetti et al., 2002, 2004, 2007; Scelfo et al., 2008) and reward (Wagner et al., 2017; Heffley and Hull, 2019; Kostadinov et al., 2019; Tsutsumi et al., 2019), which lends to the hypothesis that cerebellum supports and participates in cognitive and emotional functions, but cerebellar catecholamines have not been directly tied to these. It is these functions that are hypothesized to be aberrant in many mental illnesses, including ADHD and schizophrenia (Berridge and Waterhouse, 2003; Kapur, 2003; Heinz and Schlagenhauf, 2010; Schultz, 2013). NE is hypothesized to signal several types of information, including unexpected uncertainty and task-related utility (Aston-Jones and Cohen, 2005; Yu and Dayan, 2005). DA and NE are integral to several cognitive domains, including attention, working memory, episodic memory, social cognition, and behavioral flexibility. Accordingly, several drug classes, based on their modulation of specific components of catecholamine neurotransmission, have been developed or are proposed for the treatment of cognitive impairments in psychiatric disorders, including D1R agonists, D3R antagonists, Alpha 2A adrenergic receptor agonists and antagonists, norepinephrine transporter blockers, and catechol-O-methyltransferase inhibitors (Millan et al., 2012). In our study, the deletion of PC-derived Th demonstrates a role for it in modulating aspects of learning in appetitive, aversive, and social learning domains.

Th is found in fibers projecting throughout the cerebellar cortex and cerebellar nuclei in mice (Nelson et al., 1997). Each DA receptor subtype (D1R, D2R, D3R, D4R, and D5R) is found in the cerebellar cortex, mostly in Purkinje cell but also other layers, with varying expression in different lobules (Barili et al., 2000; Khan et al., 2000; Kim et al., 2009). Extrinsic and intrinsic sources of cerebellar catecholamines have previously been identified in multiple mammalian species (Hess and Wilson, 1991; Takada et al., 1993; Fujii et al., 1994; Abbott et al., 1996; Nelson et al., 1997; Melchitzky and Lewis, 2000). Well-differentiated dopaminergic and noradrenergic fibers innervate the cerebellar cortex in non-human primates (although their sources are unknown; Melchitzky and Lewis, 2000). Our results show that much of the Th found in the cerebellum arises from Purkinje cells. However, it is likely that much of the Th+ puncta we saw in the granular cell layer (Figure 1C) arise from LC projections. Projections from locus coeruleus to the cerebellar cortex and nuclei in rodents have long been recognized and are estimated to be the fourth largest extrinsic neuronal innervation of the cerebellum (Schweighofer et al., 2004) after mossy fibers from the basilar pons, climbing fibers from the inferior olive and serotonergic fibers from the medullary and pontine reticular formation (Hökfelt and Fuxe, 1969; Olson and Fuxe, 1971; Landis and Bloom, 1975; Landis et al., 1975; Loughlin et al., 1986a,b; Nelson et al., 1997). Despite these findings, several questions remain regarding specific sources, roles, and interactions between these catecholaminergic inputs in specific cerebellar regions and on varying cerebellar-dependent behaviors.

Although we have not established the identity of the catecholamine released by Th+ PCs in this study, or if they release catecholamines in the cerebellar nuclei, a previous report demonstrated that PCs in the posterior vermis exhibit autocrine and paracrine release of DA (Kim et al., 2009). PC-derived DA signaling is required for depolarization-induced slow current in PCs, which is triggered by calcium influx, and may play a role in regulating both pre- and post-synaptic plasticity (Kim et al., 2009). Notably, PCs in the posterior vermis also express the DA transporter and the vesicular monoamine transporter-2 (Kim et al., 2009); their spatial distribution suggests that they likely are the same as Th+ PCs we describe in this study. We showed that knockout of Th in PCs also results in decreased Th expression in LCN, suggesting that axon terminal release of catecholamine from PCs is theoretically possible. Our findings in wildtype mice confirm previous reports in mutant mouse strains that Th+ PCs co-express Aldoc (Abbott et al., 1996; Jeong et al., 2001). Differences in Aldoc staining is associated with different responses to both simple spikes and complex spikes in different functional modules of the cerebellum, with possible consequences for connections between PCs and neurons in the cerebellar nuclei (e.g., LTD vs. LTP, phase-locking vs. rebound excitation; Zhou et al., 2015; Tang et al., 2017), which may, in turn, be related to whether or not PCs express Th and release DA. PCs that are distinguished by Aldoc expression have different cerebellar nuclear projections than those that do not (Sugihara, 2011). We have previously shown that D1R+ cells exist in the LCN and modulate many cognitive behaviors (Locke et al., 2018); if DA is released from Th+ PC axon terminals, and D1R+ cells are postsynaptic to these, then this form of signaling may represent a novel and specific downstream circuit that goes beyond traditional GABAergic PC neurotransmission and may be critical in supporting cognitive functions that involve the cerebellum.

Curiously, it appears that ectopic Th expression can occur both in specific mouse mutants that are ataxic (Sawada et al., 1999a,b, 2010; White et al., 2016), and in wild type mice under hyperosmotic stress (Sakai et al., 1995). Baseline Th expression in the cerebellum also appears to be variable; in one Allen Mouse Brain Atlas experiment we examined (Table 1, Experiment #79804565), sections from a male in a 129S1SvlmJ background strain had less than half of countable Th+ PCs seen in other experiments. Parkinson’s disease is associated with reduced expression of Th, D1R, and D3R in cerebellar lobules IX and X in humans (Hurley et al., 2003). Furthermore, it appears that Th expression precedes ataxia in one strain of ataxic mice (Sawada et al., 1999a). Ectopic Th expression in PCs is also observed in calcium channel mutations (Fureman et al., 1999, 2003; Jeong et al., 2001; Sawada et al., 2010), and PC-specific knockout of Vgat (White et al., 2014). Calcium channels are associated with many neuropsychiatric illnesses and regulate aspects of both presynaptic and postsynaptic plasticity (Nanou and Catterall, 2018). While ataxias and PD are classified as movement disorders, they can be associated with prominent cognitive deficits as well (Bürk et al., 1999, 2001, 2003; Braak et al., 2005; Aarsland et al., 2010). Thus, it may be possible that increases or decreases in Th expression confer a plastic response to certain conditions or stressors. Additionally, the pattern of ectopic expression seen in ataxic models appears to be mediated by calcium and seems to maintain some zonal patterning (Sawada et al., 1999a; White et al., 2016). Most reports in ataxic mouse models show upregulation of Th expression throughout the vermis, including in the anterior lobes (Sawada et al., 1999a), though one report also notes increased Th expression throughout the hemispheres, flocculus and paraflocculus (White et al., 2016). One possibility is that Th+ PCs confer additional plasticity not seen in Th PCs that is adaptive for different environmental or pathological stressors, or that ectopic Th expression is a compensatory response to homeostatic stress (or some systemic indicator that something is wrong), which the cerebellum then tries to help correct by boosting its Th protein level. For example, Th expression is increased in expression in response to sound exposure in lateral olivocochlear (LOC) efferent neurons, which appears to be an adaptive response to changes in auditory nerve firing to possibly protect hair cell synapses from noise damage (Wu et al., 2020). On the other hand, increases in Th expression may be associated with negative consequences as well: spinocerebellar ataxias are associated with increased Th expression in the substantia nigra, which may confer risk for psychotic symptoms in these patients (Turk et al., 2018). Further experiments will be needed to test these ideas, as well as to explore the necessity and sufficiency of subregional Th expression in PCs for certain behaviors, or whether these PCs are capable of catecholamine release in the cerebellar nuclei.

**Table 1:** Allen mouse brain atlas data from six brains with *in situ* hybridization (ISH) for Tyrosine hydroxylase (Th).

Experiment number	Age	Sex	Strain	Section orientation	Total # of sections	Total # of Th+ cells
100038912	P28	M	C57/B6	Sagittal	20	2,264
1058	P56	M	C57/B6	Sagittal	20	3,182
80253685	P56	F	C57/B6	Sagittal	24	3,993
79804565	P56	M	129S1SvlmJ	Sagittal	24	1,444
100091343	18 months	M	C57/B6	Sagittal	16	2,346
100085038	24 months	M	C57/B6	Sagittal	19	3,080

## Data Availability Statement

The raw data supporting the conclusions of this article will be made available by the authors, without undue reservation.

## Ethics Statement

The animal study was reviewed and approved by the University of Washington Institutional Animal Care and Use Committee which approved all experimental protocols (4249-01) and Johns Hopkins University Animal Care and Use Committee (MO16M464).

## Author Contributions

EC, TL, and LZ designed all experiments related to *Pcp2-Cre;Th^lox/lox^* mice. HF designed Aldoc/Th double staining. EC, HF, SL, MD, and LZ wrote the manuscript. EC, AH, SJ, and TL performed behavioral experiments and analyses. EC and HF performed immunohistochemical analyses. AH performed Western Blots.

## Conflict of Interest

The authors declare that the research was conducted in the absence of any commercial or financial relationships that could be construed as a potential conflict of interest.

## References

[B1] Aarsland D. Bronnick K. Williams-Gray C. Weintraub D. Marder K. Kulisevsky J.. (2010). Mild cognitive impairment in Parkinson disease: a multicenter pooled analysis. Neurology 75, 1062–1069. 10.1212/wnl.0b013e3181f39d0e 20855849 PMC2942065

[B2] Abbott L. C. Isaacs K. R. Heckroth J. A. (1996). Co-localization of tyrosine hydroxylase and zebrin II immunoreactivities in Purkinje cells of the mutant mice, tottering and tottering/leaner. Neuroscience 71, 461–475. 10.1016/0306-4522(95)00444-0 9053800

[B3] Apps R. Hawkes R. (2009). Cerebellar cortical organization: a one-map hypothesis. Nat. Rev. Neurosci. 10, 670–681. 10.1038/nrn2698 19693030

[B4] Apps R. Hawkes R. Aoki S. Bengtsson F. Brown A. M. Chen G.. (2018). Cerebellar modules and their role as operational cerebellar processing units: a consensus paper [corrected]. Cerebellum 17, 654–682. 10.1007/s12311-018-0959-9 29876802 PMC6132822

[B6] Arnsten A. F. (2006). Stimulants: therapeutic actions in ADHD. Neuropsychopharmacology 31, 2376–2383. 10.1038/sj.npp.1301164 16855530

[B7] Arnsten A. F. Li B. M. (2005). Neurobiology of executive functions: catecholamine influences on prefrontal cortical functions. Biol. Psychiatry 57, 1377–1384. 10.1016/j.biopsych.2004.08.019 15950011

[B9] Aston-Jones G. Cohen J. D. (2005). An integrative theory of locus coeruleus-norepinephrine function: adaptive gain and optimal performance. Annu. Rev. Neurosci. 28, 403–450. 10.1146/annurev.neuro.28.061604.135709 16022602

[B8] Aston-Jones G. Waterhouse B. (2016). Locus coeruleus: from global projection system to adaptive regulation of behavior. Brain Res. 1645, 75–78. 10.1016/j.brainres.2016.03.001 26969408 PMC4969192

[B10] Balsters J. H. Cussans E. Diedrichsen J. Phillips K. A. Preuss T. M. Rilling J. K.. (2010). Evolution of the cerebellar cortex: the selective expansion of prefrontal-projecting cerebellar lobules. NeuroImage 49, 2045–2052. 10.1016/j.neuroimage.2009.10.045 19857577 PMC6436533

[B11] Barik S. de Beaurepaire R. (1996). Evidence for a functional role of the dopamine D3 receptors in the cerebellum. Brain Res. 737, 347–350. 10.1016/0006-8993(96)00964-x 8930390

[B12] Barik S. de Beaurepaire R. (2005). Dopamine D3 modulation of locomotor activity and sleep in the nucleus accumbens and in lobules 9 and 10 of the cerebellum in the rat. Prog. Neuropsychopharmacol. Biol. Psychiatry 29, 718–726. 10.1016/j.pnpbp.2005.04.020 15913875

[B13] Barili P. Bronzetti E. Ricci A. Zaccheo D. Amenta F. (2000). Microanatomical localization of dopamine receptor protein immunoreactivity in the rat cerebellar cortex. Brain Res. 854, 130–138. 10.1016/s0006-8993(99)02306-9 10784114

[B14] Bauman M. L. Kemper T. L. (2005). Neuroanatomic observations of the brain in autism: a review and future directions. Int. J. Dev. Neurosci. 23, 183–187. 10.1016/j.ijdevneu.2004.09.006 15749244

[B15] Beas B. S. McQuail J. A. Ban Uelos C. Setlow B. Bizon J. L. (2017). Prefrontal cortical GABAergic signaling and impaired behavioral flexibility in aged F344 rats. Neuroscience 345, 274–286. 10.1016/j.neuroscience.2016.02.014 26873002 PMC5333995

[B17] Berridge C. W. Devilbiss D. M. Andrzejewski M. E. Arnsten A. F. Kelley A. E. Schmeichel B.. (2006). Methylphenidate preferentially increases catecholamine neurotransmission within the prefrontal cortex at low doses that enhance cognitive function. Biol. Psychiatry 60, 1111–1120. 10.1016/j.biopsych.2006.04.022 16806100

[B16] Berridge C. W. Waterhouse B. D. (2003). The locus coeruleus-noradrenergic system: modulation of behavioral state and state-dependent cognitive processes. Brain Res. Brain Res. Rev. 42, 33–84. 10.1016/s0165-0173(03)00143-7 12668290

[B18] Bloom F. E. Hoffer B. J. Siggins G. R. (1971). Studies on norepinephrine-containing afferents to Purkinje cells of art cerebellum. I. Localization of the fibers and their synapses. Brain Res. 25, 501–521. 10.1016/0006-8993(71)90457-4 5544323

[B19] Braak H. Rub U. Jansen Steur E. N. Del Tredici K. de Vos R. A. (2005). Cognitive status correlates with neuropathologic stage in Parkinson disease. Neurology 64, 1404–1410. 10.1212/01.wnl.0000158422.41380.82 15851731

[B20] Buckner R. L. Krienen F. M. Castellanos A. Diaz J. C. Yeo B. T. (2011). The organization of the human cerebellum estimated by intrinsic functional connectivity. J. Neurophysiol. 106, 2322–2345. 10.1152/jn.00339.2011 21795627 PMC3214121

[B23] Bürk K. Bösch S. Globas C. Zühlke C. Daum I. Klockgether T.. (2001). Executive dysfunction in spinocerebellar ataxia type 1. Eur. Neurol. 46, 43–48. 10.1159/000050755 11455183

[B21] Bürk K. Globas C. Bösch S. Gräber S. Abele M. Brice A.. (1999). Cognitive deficits in spinocerebellar ataxia 2. Brain 122, 769–777. 10.1093/brain/122.4.769 10219787

[B22] Bürk K. Globas C. Bösch S. Klockgether T. Zühlke C. Daum I.. (2003). Cognitive deficits in spinocerebellar ataxia type 1, 2, and 3. J. Neurol. 250, 207–211. 10.1007/s00415-003-0976-5 12574952

[B24] Carta I. Chen C. H. Schott A. L. Dorizan S. Khodakhah K. (2019). Cerebellar modulation of the reward circuitry and social behavior. Science 363:eaav0581. 10.1126/science.aav0581 30655412 PMC6711161

[B25] Chaki S. Kawashima N. Suzuki Y. Shimazaki T. Okuyama S. (2003). Cocaine- and amphetamine-regulated transcript peptide produces anxiety-like behavior in rodents. Eur. J. Pharmacol. 464, 49–54. 10.1016/s0014-2999(03)01368-2 12600694

[B26] Couceyro P. R. Evans C. McKinzie A. Mitchell D. Dube M. Hagshenas L.. (2005). Cocaine- and amphetamine-regulated transcript (CART) peptides modulate the locomotor and motivational properties of psychostimulants. J. Pharmacol. Exp. Ther. 315, 1091–1100. 10.1124/jpet.105.091678 16099925

[B27] Freedman M. (1994). Frontal and parietal lobe dysfunction in depression: delayed alternation and tactile learning deficits. Neuropsychologia 32, 1015–1025. 10.1016/0028-3932(94)90050-7 7969863

[B30] Freedman R. (1977). Interactions of antipsychotic drugs with norepinephrine and cerebellar neuronal circuitry: implications for the psychobiology of psychosis. Biol. Psychiatry 12, 181–197. 558002

[B31] Freedman R. Hoffer B. J. Woodward D. J. Puro D. (1977). Interaction of norepinephrine with cerebellar activity evoked by mossy and climbing fibers. Exp. Neurol. 55, 269–288. 10.1016/0014-4886(77)90175-3 849758

[B28] Freedman M. Oscar-Berman M. (1986a). Bilateral frontal lobe disease and selective delayed response deficits in humans. Behav. Neurosci. 100, 337–342. 10.1037/0735-7044.100.3.337 3730139

[B29] Freedman M. Oscar-Berman M. (1986b). Selective delayed response deficits in Parkinson’s and Alzheimer’s disease. Arch. Neurol. 43, 886–890. 10.1001/archneur.1986.00520090026011 3741206

[B32] Fujii T. Sakai M. Nagatsu I. (1994). Immunohistochemical demonstration of expression of tyrosine hydroxylase in cerebellar Purkinje cells of the human and mouse. Neurosci. Lett. 165, 161–163. 10.1016/0304-3940(94)90734-x 7912416

[B33] Fujita H. Aoki H. Ajioka I. Yamazaki M. Abe M. Oh-Nishi A.. (2014). Detailed expression pattern of aldolase C (Aldoc) in the cerebellum, retina and other areas of the CNS studied in Aldoc-Venus knock-in mice. PLoS One 9:e86679. 10.1371/journal.pone.0086679 24475166 PMC3903578

[B34] Fujita H. Morita N. Furuichi T. Sugihara I. (2012). Clustered fine compartmentalization of the mouse embryonic cerebellar cortex and its rearrangement into the postnatal striped configuration. J. Neurosci. 32, 15688–15703. 10.1523/JNEUROSCI.1710-12.2012 23136409 PMC6621621

[B35] Fureman B. E. Campbell D. B. Hess E. J. (1999). L-type calcium channel regulation of abnormal tyrosine hydroxylase expression in cerebella of tottering mice. Ann. N. Y. Acad. Sci. 868, 217–219. 10.1111/j.1749-6632.1999.tb11289.x 10414297

[B36] Fureman B. E. Campbell D. B. Hess E. J. (2003). Regulation of tyrosine hydroxylase expression in tottering mouse Purkinje cells. Neurotox Res. 5, 521–528. 10.1007/BF03033162 14715436

[B37] Gore B. B. Zweifel L. S. (2013). Genetic reconstruction of dopamine D1 receptor signaling in the nucleus accumbens facilitates natural and drug reward responses. J. Neurosci. 33, 8640–8649. 10.1523/JNEUROSCI.5532-12.2013 23678109 PMC3684445

[B38] Harley C. W. (2004). Norepinephrine and dopamine as learning signals. Neural Plast. 11, 191–204. 10.1155/np.2004.191 15656268 PMC2567044

[B39] Harrison P. W. Montgomery S. H. (2017). Genetics of cerebellar and neocortical expansion in anthropoid primates: a comparative approach. Brain Behav. Evol. 89, 274–285. 10.1159/000477432 28683440 PMC5637284

[B40] Heffley W. Hull C. (2019). Classical conditioning drives learned reward prediction signals in climbing fibers across the lateral cerebellum. Elife 8:e46764. 10.7554/elife.46764 31509108 PMC6845228

[B41] Heinz A. Schlagenhauf F. (2010). Dopaminergic dysfunction in schizophrenia: salience attribution revisited. Schizophr. Bull. 36, 472–485. 10.1093/schbul/sbq031 20453041 PMC2879696

[B42] Hess E. J. Wilson M. C. (1991). Tottering and leaner mutations perturb transient developmental expression of tyrosine hydroxylase in embryologically distinct Purkinje cells. Neuron 6, 123–132. 10.1016/0896-6273(91)90127-l 1670919

[B43] Hökfelt T. Fuxe K. (1969). Cerebellar monoamine nerve terminals, a new type of afferent fibers to the cortex cerebelli. Exp. Brain Res. 9, 63–72. 10.1007/bf00235452 5808481

[B44] Hurley M. J. Mash D. C. Jenner P. (2003). Markers for dopaminergic neurotransmission in the cerebellum in normal individuals and patients with Parkinson’s disease examined by RT-PCR. Eur. J. Neurosci. 18, 2668–2672. 10.1046/j.1460-9568.2003.02963.x 14622169

[B45] Itō M. (1984). The Cerebellum and Neural Control. New York, NY: Raven Press.

[B46] Jackson C. R. Ruan G. X. Aseem F. Abey J. Gamble K. Stanwood G.. (2012). Retinal dopamine mediates multiple dimensions of light-adapted vision. J. Neurosci. 32, 9359–9368. 10.1523/JNEUROSCI.0711-12.2012 22764243 PMC3400466

[B47] Jeong Y. G. Kim M. K. Hawkes R. (2001). Ectopic expression of tyrosine hydroxylase in Zebrin II immunoreactive Purkinje cells in the cerebellum of the ataxic mutant mouse, pogo. Brain Res. Dev. Brain Res. 129, 201–209. 10.1016/s0165-3806(01)00212-7 11506864

[B48] Kapur S. (2003). Psychosis as a state of aberrant salience: a framework linking biology, phenomenology, and pharmacology in schizophrenia. Am. J. Psychiatry 160, 13–23. 10.1176/appi.ajp.160.1.13 12505794

[B49] Kelly R. M. Strick P. L. (2003). Cerebellar loops with motor cortex and prefrontal cortex of a nonhuman primate. J. Neurosci. 23, 8432–8444. 10.1523/JNEUROSCI.23-23-08432.2003 12968006 PMC6740694

[B50] Khan Z. U. Gutiérrez A. Martín R. Peñafiel A. Rivera A. de la Calle A. (2000). Dopamine D5 receptors of rat and human brain. Neuroscience 100, 689–699. 10.1016/s0306-4522(00)00274-8 11036203

[B51] Kim Y. S. Shin J. H. Hall F. S. Linden D. J. (2009). Dopamine signaling is required for depolarization-induced slow current in cerebellar Purkinje cells. J. Neurosci. 29, 8530–8538. 10.1523/JNEUROSCI.0468-09.2009 19571144 PMC2720617

[B52] Kostadinov D. Beau M. Blanco-Pozo M. Hausser M. (2019). Predictive and reactive reward signals conveyed by climbing fiber inputs to cerebellar Purkinje cells. Nat. Neurosci. 22, 950–962. 10.1038/s41593-019-0381-8 31036947 PMC7612392

[B53] Laatikainen L. M. Sharp T. Harrison P. J. Tunbridge E. M. (2013). Sexually dimorphic effects of catechol-O-methyltransferase (COMT) inhibition on dopamine metabolism in multiple brain regions. PLoS One 8:e61839. 10.1371/journal.pone.0061839 23613951 PMC3629045

[B54] Landis S. C. Bloom F. E. (1975). Ultrastructural identification of noradrenergic boutons in mutant and normal mouse cerebellar cortex. Brain Res. 96, 299–305. 10.1016/0006-8993(75)90738-6 1175014

[B55] Landis S. C. Shoemaker W. J. Schlumpf M. Bloom F. E. (1975). Catecholamines in mutant mouse cerebellum: fluorescence microscopic and chemical studies. Brain Res. 93, 253–266. 10.1016/0006-8993(75)90349-2 1174970

[B57] Lein E. S. Hawrylycz M. J. Ao N. Ayres M. Bensinger A. Bernard A.. (2007). Genome-wide atlas of gene expression in the adult mouse brain. Nature 445, 168–176. 10.1038/nature05453 17151600

[B58] Leto K. Arancillo M. Becker E. B. Buffo A. Chiang C. Ding B.. (2016). Consensus paper: cerebellar development. Cerebellum 15, 789–828. 10.1007/s12311-015-0724-2 26439486 PMC4846577

[B59] Levant B. DeSouza E. B. (1993). Differential pharmacological profile of striatal and cerebellar dopamine receptors labeled by [^3^H]quinpirole: identification of a discrete population of putative D3 receptors. Synapse 14, 90–95. 10.1002/syn.890140112 8099762

[B60] Locke T. M. Soden M. E. Miller S. M. Hunker A. Knakal C. Licholai J. A.. (2018). Dopamine D_1_ receptor-positive neurons in the lateral nucleus of the cerebellum contribute to cognitive behavior. Biol. Psychiatry 84, 401–412. 10.1016/j.biopsych.2018.01.019 29478701 PMC6072628

[B61] Loughlin S. E. Foote S. L. Bloom F. E. (1986a). Efferent projections of nucleus locus coeruleus: topographic organization of cells of origin demonstrated by three-dimensional reconstruction. Neuroscience 18, 291–306. 10.1016/0306-4522(86)90155-7 3736860

[B62] Loughlin S. E. Foote S. L. Grzanna R. (1986b). Efferent projections of nucleus locus coeruleus: morphologic subpopulations have different efferent targets. Neuroscience 18, 307–319. 10.1016/0306-4522(86)90156-9 3736861

[B63] Loveland K. A. Bachevalier J. Pearson D. A. Lane D. M. (2008). Fronto-limbic functioning in children and adolescents with and without autism. Neuropsychologia 46, 49–62. 10.1016/j.neuropsychologia.2007.08.017 17936314 PMC2785231

[B64] Luo Y. Fujita H. Nedelescu H. Biswas M. S. Sato C. Ying S.. (2017). Lobular homology in cerebellar hemispheres of humans, non-human primates and rodents: a structural, axonal tracing and molecular expression analysis. Brain Struct. Funct. 222, 2449–2472. 10.1007/s00429-017-1436-9 28508291

[B65] McCormick D. A. Thompson R. F. (1982). Locus coeruleus lesions and resistance to extinction of a classically conditioned response: involvement of the neocortex and hippocampus. Brain Res. 245, 239–249. 10.1016/0006-8993(82)90806-x 7127072

[B66] McElligott J. G. Freedman W. (1988). Vestibulo-ocular reflex adaptation in cats before and after depletion of norepinephrine. Exp. Brain Res. 69, 509–521. 10.1007/bf00247305 3131154

[B67] Melchitzky D. S. Lewis D. A. (2000). Tyrosine hydroxylase- and dopamine transporter-immunoreactive axons in the primate cerebellum. Evidence for a lobular- and laminar-specific dopamine innervation. Neuropsychopharmacology 22, 466–472. 10.1016/s0893-133x(99)00139-6 10731621

[B68] Millan M. J. Agid Y. Brune M. Bullmore E. T. Carter C. S. Clayton N. S.. (2012). Cognitive dysfunction in psychiatric disorders: characteristics, causes and the quest for improved therapy. Nat. Rev. Drug Discov. 11, 141–168. 10.1038/nrd3628 22293568

[B69] Nanou E. Catterall W. A. (2018). Calcium channels, synaptic plasticity, and neuropsychiatric disease. Neuron 98, 466–481. 10.1016/j.neuron.2018.03.017 29723500

[B70] Nautiyal K. M. Tanaka K. F. Barr M. M. Tritschler L. Le Dantec Y. David D. J.. (2015). Distinct circuits underlie the effects of 5-HT1B receptors on aggression and impulsivity. Neuron 86, 813–826. 10.1016/j.neuron.2015.03.041 25892302 PMC4431594

[B71] Nelson T. E. King J. S. Bishop G. A. (1997). Distribution of tyrosine hydroxylase-immunoreactive afferents to the cerebellum differs between species. J. Comp. Neurol. 379, 443–454. 10.1002/(sici)1096-9861(19970317)379:3<443::aid-cne9>3.0.co;2-3 9067835

[B72] Olson L. Fuxe K. (1971). On the projections from the locus coeruleus noradrealine neurons: the cerebellar innervation. Brain Res. 28, 165–171. 10.1016/0006-8993(71)90533-6 4104275

[B73] O’Reilly J. X. Mesulam M. M. Nobre A. C. (2008). The cerebellum predicts the timing of perceptual events. J. Neurosci. 28, 2252–2260. 10.1523/jneurosci.2742-07.2008 18305258 PMC6671847

[B74] Oscarsson O. (1979). Functional units of the cerebellum - sagittal zones and microzones. Trends Neurosci. 2, 143–145. 10.1016/0166-2236(79)90057-2

[B75] Panagopoulos N. T. Papadopoulos G. C. Matsokis N. A. (1991). Dopaminergic innervation and binding in the rat cerebellum. Neurosci. Lett. 130, 208–212. 10.1016/0304-3940(91)90398-d 1795884

[B76] Paredes D. A. Cartford M. C. Catlow B. J. Samec A. Avilas M. George A.. (2009). Neurotransmitter release during delay eyeblink classical conditioning: role of norepinephrine in consolidation and effect of age. Neurobiol. Learn. Mem. 92, 267–282. 10.1016/j.nlm.2008.08.008 18809505 PMC2752948

[B77] Park S. Holzman P. S. (1992). Schizophrenics show spatial working memory deficits. Arch. Gen. Psychiatry 49, 975–982. 10.1001/archpsyc.1992.01820120063009 1449384

[B78] Parker K. L. Kim Y. C. Kelley R. M. Nessler A. J. Chen K. H. Muller-Ewald V. A.. (2017). Delta-frequency stimulation of cerebellar projections can compensate for schizophrenia-related medial frontal dysfunction. Mol. Psychiatry 22, 647–655. 10.1038/mp.2017.50 28348382 PMC5873945

[B79] Penn H. E. (2006). Neurobiological correlates of autism: a review of recent research. Child Neuropsychol. 12, 57–79. 10.1080/09297040500253546 16484102

[B80] Reeber S. L. Sillitoe R. V. (2011). Patterned expression of a cocaine- and amphetamine-regulated transcript peptide reveals complex circuit topography in the rodent cerebellar cortex. J. Comp. Neurol. 519, 1781–1796. 10.1002/cne.22601 21452228

[B81] Rogers T. D. Dickson P. E. Heck D. H. Goldowitz D. Mittleman G. Blaha C. D. (2011). Connecting the dots of the cerebro-cerebellar role in cognitive function: neuronal pathways for cerebellar modulation of dopamine release in the prefrontal cortex. Synapse 65, 1204–1212. 10.1002/syn.20960 21638338 PMC3854794

[B82] Rossi M. A. Hayrapetyan V. Y. Maimon B. Mak K. Je H. S. Yin H. H. (2012). Prefrontal cortical mechanisms underlying delayed alternation in mice. J. Neurophysiol. 108, 1211–1222. 10.1152/jn.01060.2011 22539827

[B83] Russo-Neustadt A. Cotman C. W. (1997). Adrenergic receptors in Alzheimer’s disease brain: selective increases in the cerebella of aggressive patients. J. Neurosci. 17, 5573–5580. 10.1523/jneurosci.17-14-05573.1997 9204938 PMC6793809

[B84] Russo-Neustadt A. Zomorodian T. J. Cotman C. W. (1998). Preserved cerebellar tyrosine hydroxylase-immunoreactive neuronal fibers in a behaviorally aggressive subgroup of Alzheimer’s disease patients. Neuroscience 87, 55–61. 10.1016/s0306-4522(98)00134-1 9722141

[B86] Sacchetti B. Baldi E. Lorenzini C. A. Bucherelli C. (2002). Cerebellar role in fear-conditioning consolidation. Proc. Natl. Acad. Sci. U S A 99, 8406–8411. 10.1073/pnas.112660399 12034877 PMC123080

[B87] Sacchetti B. Sacco T. Strata P. (2007). Reversible inactivation of amygdala and cerebellum but not perirhinal cortex impairs reactivated fear memories. Eur. J. Neurosci. 25, 2875–2884. 10.1111/j.1460-9568.2007.05508.x 17466022

[B85] Sacchetti B. Scelfo B. Tempia F. Strata P. (2004). Long-term synaptic changes induced in the cerebellar cortex by fear conditioning. Neuron 42, 973–982. 10.1016/j.neuron.2004.05.012 15207241

[B88] Sakai M. Fujii T. Karasawa N. Arai R. Nagatsu I. (1995). Enhanced expression of tyrosine hydroxylase and aromatic L-amino acid decarboxylase in cerebellar Purkinje cells of mouse after hyperosmotic stimuli. Neurosci. Lett. 194, 142–144. 10.1016/0304-3940(95)11716-a 7478200

[B89] Sanford C. A. Soden M. E. Baird M. A. Miller S. M. Schulkin J. Palmiter R. D.. (2017). A central amygdala CRF circuit facilitates learning about weak threats. Neuron 93, 164–178. 10.1016/j.neuron.2016.11.034 28017470 PMC5217711

[B91] Sawada K. Sakata-Haga H. Fukui Y. (2010). Alternating array of tyrosine hydroxylase and heat shock protein 25 immunopositive Purkinje cell stripes in zebrin II-defined transverse zone of the cerebellum of rolling mouse Nagoya. Brain Res. 1343, 46–53. 10.1016/j.brainres.2010.04.062 20462503

[B92] Sawada K. Komatsu S. Haga H. Oda S. Fukui Y. (1999a). Abnormal expression of tyrosine hydroxylase immunoreactivity in Purkinje cells precedes the onset of ataxia in dilute-lethal mice. Brain Res. 844, 188–191. 10.1016/s0006-8993(99)01899-5 10536275

[B93] Sawada K. Komatsu S. Haga H. Sun X. Z. Hisano S. Fukui Y. (1999b). Abnormal expression of tyrosine hydroxylase immunoreactivity in cerebellar cortex of ataxic mutant mice. Brain Res. 829, 107–112. 10.1016/s0006-8993(99)01347-5 10350535

[B94] Scelfo B. Sacchetti B. Strata P. (2008). Learning-related long-term potentiation of inhibitory synapses in the cerebellar cortex. Proc. Natl. Acad. Sci. U S A 105, 769–774. 10.1073/pnas.0706342105 18184813 PMC2206611

[B95] Schmahmann J. D. (2019). The cerebellum and cognition. Neurosci. Lett. 688, 62–75. 10.1016/j.neulet.2018.07.005 29997061

[B96] Schmahmann J. D. Guell X. Stoodley C. J. Halko M. A. (2019). The theory and neuroscience of cerebellar cognition. Annu. Rev. Neurosci. 42, 337–364. 10.1146/annurev-neuro-070918-050258 30939101

[B97] Schultz W. (2013). Updating dopamine reward signals. Curr. Opin. Neurobiol. 23, 229–238. 10.1016/j.conb.2012.11.012 23267662 PMC3866681

[B98] Schultz W. Stauffer W. R. Lak A. (2017). The phasic dopamine signal maturing: from reward *via* behavioural activation to formal economic utility. Curr. Opin. Neurobiol. 43, 139–148. 10.1016/j.conb.2017.03.013 28390863

[B99] Schweighofer N. Doya K. Kuroda S. (2004). Cerebellar aminergic neuromodulation: towards a functional understanding. Brain Res. Rev. 44, 103–116. 10.1016/j.brainresrev.2003.10.004 15003388

[B100] Silverman J. L. Yang M. Lord C. Crawley J. N. (2010). Behavioural phenotyping assays for mouse models of autism. Nat. Rev. Neurosci. 11, 490–502. 10.1038/nrn2851 20559336 PMC3087436

[B101] Sokolowski J. D. Salamone J. D. (1994). Effects of dopamine depletions in the medial prefrontal cortex on DRL performance and motor activity in the rat. Brain Res. 642, 20–28. 10.1016/0006-8993(94)90901-6 8032881

[B102] Stoodley C. J. Schmahmann J. D. (2009). Functional topography in the human cerebellum: a meta-analysis of neuroimaging studies. NeuroImage 44, 489–501. 10.1016/j.neuroimage.2008.08.039 18835452

[B103] Stoodley C. J. Schmahmann J. D. (2010). Evidence for topographic organization in the cerebellum of motor control versus cognitive and affective processing. Cortex 46, 831–844. 10.1016/j.cortex.2009.11.008 20152963 PMC2873095

[B104] Stoodley C. J. Valera E. M. Schmahmann J. D. (2012). Functional topography of the cerebellum for motor and cognitive tasks: an fMRI study. NeuroImage 59, 1560–1570. 10.1016/j.neuroimage.2011.08.065 21907811 PMC3230671

[B105] Sugihara I. (2011). Compartmentalization of the deep cerebellar nuclei based on afferent projections and aldolase C expression. Cerebellum 10, 449–463. 10.1007/s12311-010-0226-1 20981512

[B106] Takada M. Sugimoto T. Hattori T. (1993). Tyrosine hydroxylase immunoreactivity in cerebellar Purkinje cells of the rat. Neurosci. Lett. 150, 61–64. 10.1016/0304-3940(93)90108-w 8097025

[B107] Takeuchi T. Kiyama Y. Nakamura K. Tsujita M. Matsuda I. Mori H.. (2001). Roles of the glutamate receptor ε2 and δ2 subunits in the potentiation and prepulse inhibition of the acoustic startle reflex. Eur. J. Neurosci. 14, 153–160. 10.1046/j.0953-816x.2001.01620.x 11488959

[B108] Tang T. Xiao J. Suh C. Y. Burroughs A. Cerminara N. L. Jia L.. (2017). Heterogeneity of Purkinje cell simple spike-complex spike interactions: zebrin- and non-zebrin-related variations. J. Physiol. 595, 5341–5357. 10.1113/jp274252 28516455 PMC5538194

[B109] Tsutsumi S. Hidaka N. Isomura Y. Matsuzaki M. Sakimura K. Kano M.. (2019). Modular organization of cerebellar climbing fiber inputs during goal-directed behavior. Elife 8:e47021. 10.7554/elife.47021 31596238 PMC6844646

[B110] Turk K. W. Flanagan M. E. Josephson S. Keene C. D. Jayadev S. Bird T. D. (2018). Psychosis in spinocerebellar ataxias: a case series and study of tyrosine hydroxylase in substantia nigra. Cerebellum 17, 143–151. 10.1007/s12311-017-0882-5 28887803 PMC5843512

[B111] Upadhya M. A. Nakhate K. T. Kokare D. M. Singru P. S. Subhedar N. K. (2011). Cocaine- and amphetamine-regulated transcript peptide increases spatial learning and memory in rats. Life Sci. 88, 322–334. 10.1016/j.lfs.2010.12.008 21167182

[B112] Urs N. M. Nicholls P. J. Caron M. G. (2014). Integrated approaches to understanding antipsychotic drug action at GPCRs. Curr. Opin. Cell Biol. 27, 56–62. 10.1016/j.ceb.2013.11.002 24680431 PMC5702556

[B113] Van Overwalle F. Baetens K. Marien P. Vandekerckhove M. (2014). Social cognition and the cerebellum: a meta-analysis of over 350 fMRI studies. NeuroImage 86, 554–572. 10.1016/j.neuroimage.2013.09.033 24076206

[B114] Vermeiren Y. Van Dam D. Aerts T. Engelborghs S. De Deyn P. P. (2014a). Brain region-specific monoaminergic correlates of neuropsychiatric symptoms in Alzheimer’s disease. J. Alzheimers Dis. 41, 819–833. 10.3233/jad-140309 24685637

[B115] Vermeiren Y. Van Dam D. Aerts T. Engelborghs S. De Deyn P. P. (2014b). Monoaminergic neurotransmitter alterations in postmortem brain regions of depressed and aggressive patients with Alzheimer’s disease. Neurobiol. Aging 35, 2691–2700. 10.1016/j.neurobiolaging.2014.05.031 24997673

[B116] Versteeg D. H. Van Der Gugten J. De Jong W. Palkovits M. (1976). Regional concentrations of noradrenaline and dopamine in rat brain. Brain Res. 113, 563–574. 10.1016/0006-8993(76)90057-3 953752

[B117] Voogd J. (2014). What we do not know about cerebellar systems neuroscience. Front. Syst. Neurosci. 8:227. 10.3389/fnsys.2014.00227 25565986 PMC4270173

[B118] Voogd J. Glickstein M. (1998). The anatomy of the cerebellum. Trends Neurosci. 21, 370–375. 10.1016/s0166-2236(98)01318-6 9735944

[B119] Wagner M. J. Kim T. H. Savall J. Schnitzer M. J. Luo L. (2017). Cerebellar granule cells encode the expectation of reward. Nature 544, 96–100. 10.1038/nature21726 28321129 PMC5532014

[B122] Watson T. C. Becker N. Apps R. Jones M. W. (2014). Back to front: cerebellar connections and interactions with the prefrontal cortex. Front. Syst. Neurosci. 8:4. 10.3389/fnsys.2014.00004 24550789 PMC3912388

[B120] Watson M. McElligott J. G. (1983). 6-OHDA induced effects upon the acquisition and performance of specific locomotor tasks in rats. Pharmacol. Biochem. Behav. 18, 927–934. 10.1016/s0091-3057(83)80016-1 6412249

[B121] Watson M. McElligott J. G. (1984). Cerebellar norepinephrine depletion and impaired acquisition of specific locomotor tasks in rats. Brain Res. 296, 129–138. 10.1016/0006-8993(84)90518-3 6424867

[B123] White J. J. Arancillo M. King A. Lin T. Miterko L. N. Gebre S. A.. (2016). Pathogenesis of severe ataxia and tremor without the typical signs of neurodegeneration. Neurobiol. Dis. 86, 86–98. 10.1016/j.nbd.2015.11.008 26586559 PMC4778569

[B124] White J. J. Arancillo M. Stay T. L. George-Jones N. A. Levy S. L. Heck D. H.. (2014). Cerebellar zonal patterning relies on Purkinje cell neurotransmission. J. Neurosci. 34, 8231–8245. 10.1523/jneurosci.0122-14.2014 24920627 PMC4051975

[B125] Winsky L. Harvey J. A. (1992). 6-Hydroxydopamine induced impairment of Pavlovian conditioning in the rabbit. Neurochem. Res. 17, 415–422. 10.1007/bf00969886 1528351

[B126] Wu J. S. Yi E. Manca M. Javaid H. Lauer A. M. Glowatzki E. (2020). Sound exposure dynamically induces dopamine synthesis in cholinergic LOC efferents for feedback to auditory nerve fibers. Elife 9:e52419. 10.7554/elife.52419 31975688 PMC7043886

[B127] Yu A. J. Dayan P. (2005). Uncertainty, neuromodulation, and attention. Neuron 46, 681–692. 10.1016/j.neuron.2005.04.026 15944135

[B128] Zhang X. M. Ng A. H. Tanner J. A. Wu W. T. Copeland N. G. Jenkins N. A.. (2004). Highly restricted expression of Cre recombinase in cerebellar Purkinje cells. Genesis 40, 45–51. 10.1002/gene.20062 15354293

[B129] Zhou H. Voges K. Lin Z. Ju C. Schonewille M. (2015). Differential Purkinje cell simple spike activity and pausing behavior related to cerebellar modules. J. Neurophysiol. 113, 2524–2536. 10.1152/jn.00925.2014 25717166 PMC4416590

