# Supplementary figures and images for: Purkinje Cell-Specific Knockout of Tyrosine Hydroxylase Impairs Cognitive Behaviors
Source: Front Cell Neurosci. 2020 Jul 29;14:228. doi: 10.3389/fncel.2020.00228 (PMC7403473; doi:10.3389/fncel.2020.00228)

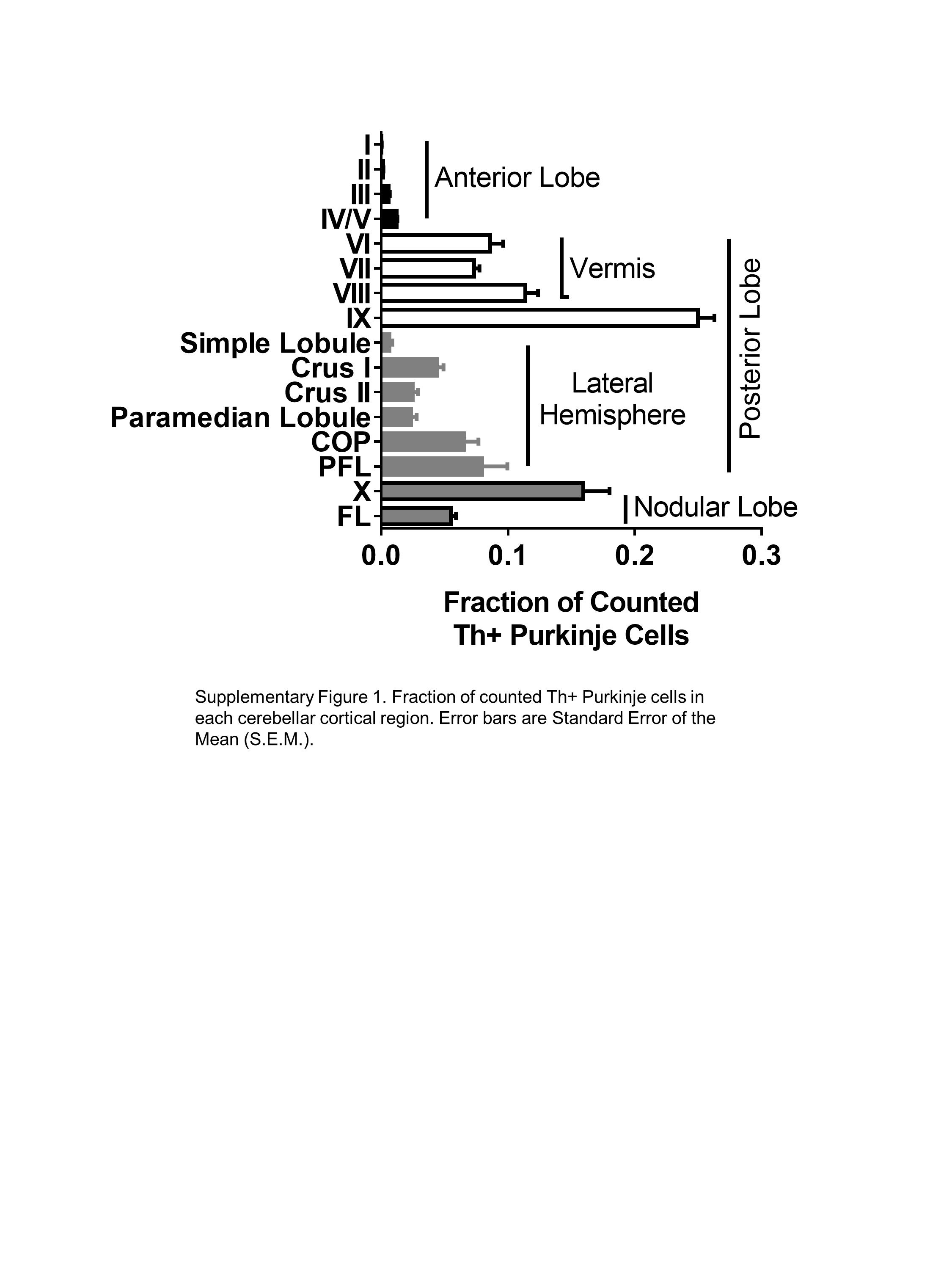

Supplement: Supplementary file 1 [file Image_1.TIF]

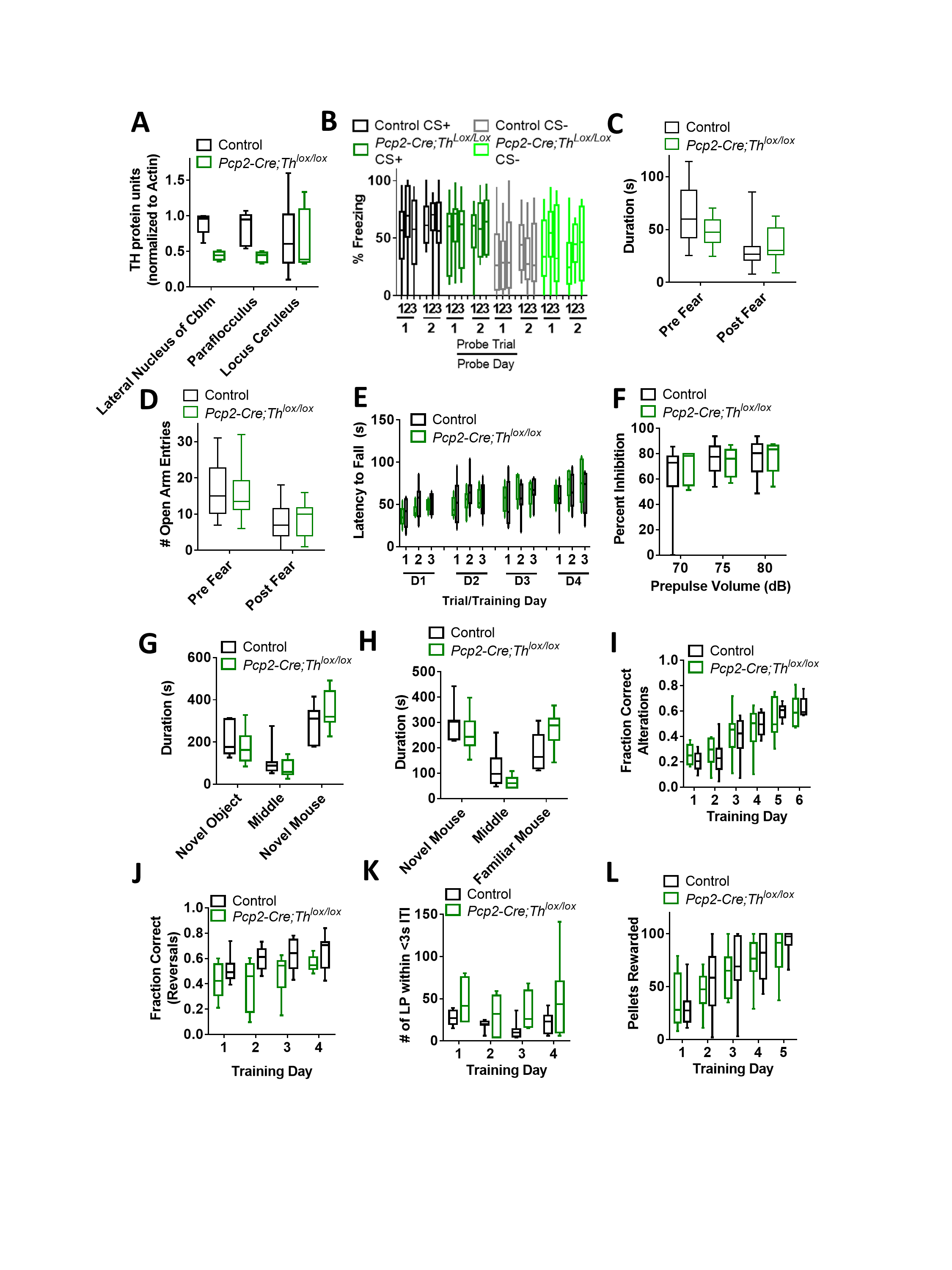

Supplement: Supplementary file 2 [file Image_2.TIF]

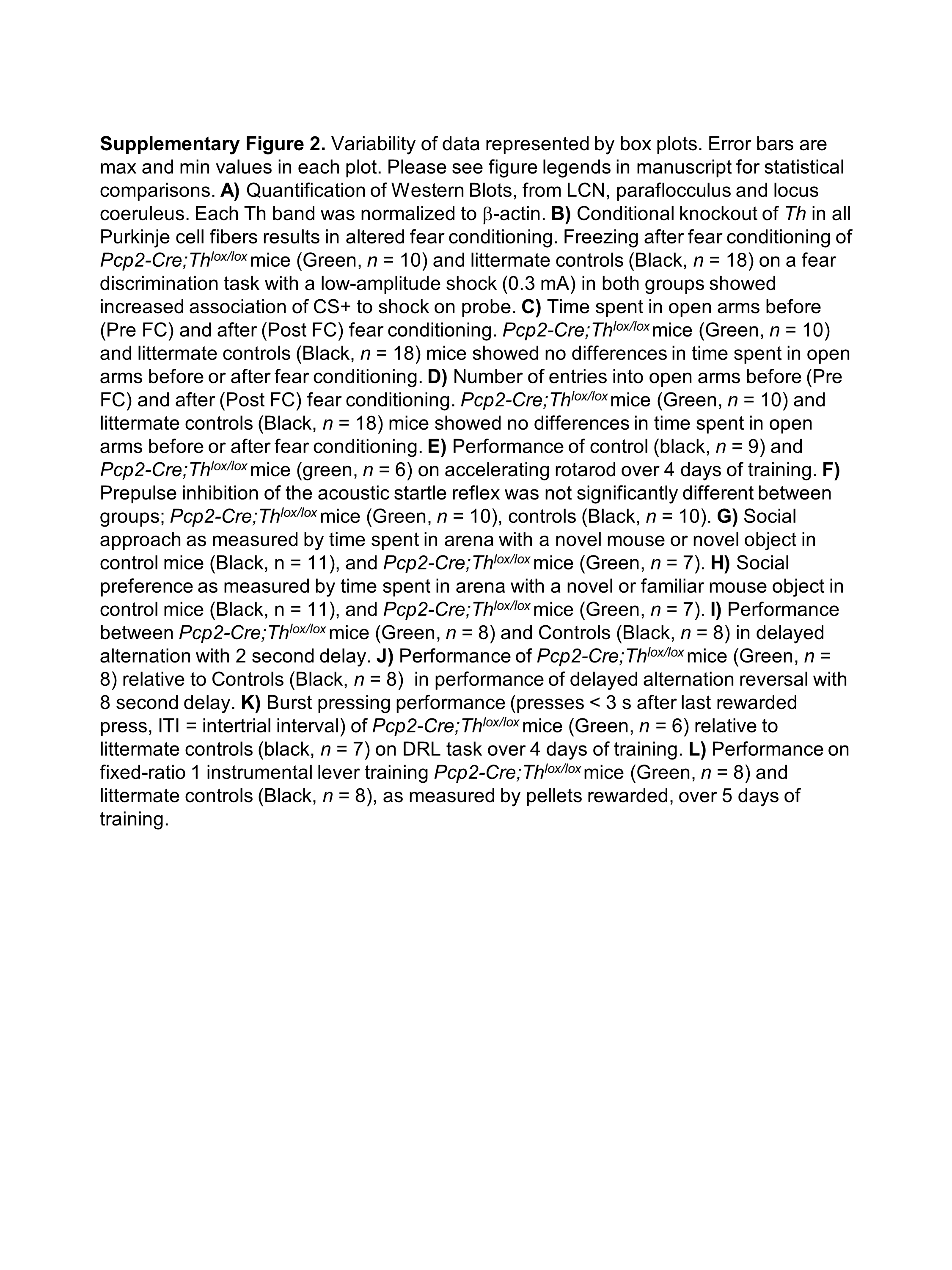

Supplement: Supplementary file 3 [file Image_3.TIF]
